# A clustering-based method for estimating pennation angle from B-mode ultrasound images

**DOI:** 10.1017/wtc.2022.30

**Published:** 2023-03-01

**Authors:** Xuefeng Bao, Qiang Zhang, Natalie Fragnito, Jian Wang, Nitin Sharma

**Affiliations:** 1 Department of Biomedical Engineering, University of Wisconsin-Milwaukee, Milwaukee, WI, USA; 2 Joint Department of Biomedical Engineering, North Carolina State University, Raleigh, NC, USA; 3 Joint Department of Biomedical Engineering, The University of North Carolina, Chapel Hill, NC, USA; 4 Snap Inc., New York, NY, USA

**Keywords:** clustering, dorsiflexion, human intent, ultrasound imaging

## Abstract

B-mode ultrasound (US) is often used to noninvasively measure skeletal muscle architecture, which contains human intent information. Extracted features from B-mode images can help improve closed-loop human–robotic interaction control when using rehabilitation/assistive devices. The traditional manual approach to inferring the muscle structural features from US images is laborious, time-consuming, and subjective among different investigators. This paper proposes a clustering-based detection method that can mimic a well-trained human expert in identifying fascicle and aponeurosis and, therefore, compute the pennation angle. The clustering-based architecture assumes that muscle fibers have tubular characteristics. It is robust for low-frequency image streams. We compared the proposed algorithm to two mature benchmark techniques: *UltraTrack* and *ImageJ.* The performance of the proposed approach showed higher accuracy in our dataset (frame frequency is 20 Hz), that is, similar to the human expert. The proposed method shows promising potential in automatic muscle fascicle orientation detection to facilitate implementations in biomechanics modeling, rehabilitation robot control design, and neuromuscular disease diagnosis with low-frequency data stream.

## 1. Introduction

The human musculoskeletal system plays an essential role in enabling various functional human limb movements in daily life, for example, sit-to-stand, walking, reaching, and grasping. Neuromuscular disorders, including spinal cord injury (SCI) and stroke, paralyze skeletal muscles, thus impairing normal activities of daily living. Muscle architectural features of the paralyzed muscles such as muscle fascicle length, fascicle orientation, pennation angle (PA), and muscle thickness can potentially reveal residual motor intent, which can be compensated effectively through neuroprosthetic or robotic intervention (Jahanandish et al., 2019; Zhang et al., 2020a). In addition, examining the skeletal muscle architectural features can also help diagnose muscle degenerative conditions, for example, sarcopenia (Mueller et al., 2016; Ticinesi et al., 2017; Chang et al., 2018).

The extraction of skeletal muscle architectural features depends on the detection of muscle fascicles (i.e., bundles of skeletal muscle fibers) and aponeuroses (i.e., connective tissues). Ultrasound (US) imaging has been widely applied to detect muscle architectural features given different tissues with different acoustic impedance. In US imaging, the tissues can be visualized as hyperechoic tubular structures (Zhao and Zhang, 2011). Compared with magnetic resonance imaging (MRI), US imaging is a low-cost, radiation-free, and time-efficient real-time display technology. Therefore, US imaging has been widely used in research and clinical studies to investigate muscle architectural features for biomechanics modeling and neuromuscular disease diagnosis. Manual detection is robust and reliable across a broad range of experimental conditions (Kwah et al., 2013). However, this traditional method is time- and energy-consuming, particularly when massive temporal sequences of US imaging frames need to be analyzed (O’Brien et al., 2010; Giannakou et al., 2011; Baroni et al., 2013). Moreover, the detection performance is relatively subjective among different investigators (Darby et al., 2012; Zhou et al., 2015).

In recent years, several semiautomatic and fully automatic muscle fascicle tracking algorithms have been proposed to efficiently and effectively extract muscle architectural features (Cronin et al., 2011; Damon et al., 2011; Gillett et al., 2013; Zhou and Zheng, 2015; Marzilger et al., 2018; Liu et al., 2019; Wang et al., 2019; Yuan et al., 2020). In Zhou et al. (2015), the authors developed a Gabor wavelet with Hough transform (GWHT) method to achieve a successful detection of PA. This method simulated human vision as the investigator selected the muscle fascicles. With prior knowledge about the distribution and shape of fascicles and aponeuroses, the correlation between the manually measured angle and the automatically detected angle could be as high as above 0.9, and the error was approximately 1.5°. However, it has a relatively long processing time (20 s with an Intel Core 2 Q8400 2.66-GHz processor) due to the complexity of the Hough transform, which still leaves room for improvement.

Marzilger et al. (2018) developed a semiautomated algorithm for measuring vastus lateralis muscle architecture, and its inter-day reliability was effectively validated. However, pixel brightness (i.e., intensity, echo intensity, or echogenicity), an important factor for selecting the target fascicle, was not considered in that paper. In addition, although the computation time for a typical video with approximately 500 frames and a region of interest (ROI) of 614×140 pixels was approximately 2 min, an additional 10–15 min period was necessary to control and adjust possible alternations within the video. Thus, its long processing and tuning time limits the fully automatic applications in real time.

A belt linear summation transform (BLST) method was derived by Wang et al. (2019) that considers the pixel brightness and does not require any extra domain knowledge. However, its time complexity, 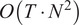 , where 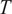 denotes the resolution of the rotation and 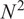 denotes the resolution of the image, is still too high. Radon transformation (RTF) is also a well-developed and applied technique for muscle fascicle orientation or PA detection from US imaging (Zhao and Zhang, 2011; Liu et al., 2019; Yuan et al., 2020). However, its complexity is higher than BLST, as proven by Wang et al. (2019).

As one of the most successful methods, the optical flow algorithms (Cronin et al., 2011; Gillett et al., 2013) have been commercialized as a Matlab toolbox *UltraTrack* (Farris and Lichtwark, 2016). This toolbox has been updated from the first generation that can only track a single muscle fascicle to the recent fourth generation that enables the tracking of multiple fascicles. The toolbox can detect and track the orientations and lengths of fascicle and aponeurosis, thus, computing the PA sequence using the orientations (Darby et al., 2012; Kawamoto et al., 2014). The core technique of *UltraTrack* is to implement affine flow algorithms and pursue features from one frame to another using Lucas–Kanade or cross-correlation approaches (Cronin et al., 2011; Darby et al., 2012; Gillett et al., 2013; Farris and Lichtwark, 2016). In general, this method requires a manual determination of the tracking features in the initial image frame. Then, the locations of key points defined in the initial image frame are automatically tracked through image frames (Van Hooren et al., 2020). In some cases, for example, when movements of the selected key points between consecutive images are not sufficiently small which is usually seen in low-frequency image frames, it may lose the good tracking (Zhou et al., 2015). Another limitation of optical flow is the drift error. But it was addressed in a recently developed method, that is, *TimTrack* (van der Zee and Kuo, 2022).

Another flagship method to measure muscle fascicle orientation or PA is *ImageJ* (Abràmoff et al., 2004; Seynnes and Cronin, 2020), which can be conveniently implemented across platforms. The consensus-based algorithm rotates the detection angle for the image and observes which one reeves the most data points, and this angle is regarded as the fascicle orientation. However, tracking accuracy cannot be guaranteed when the image contains noises.

This paper proposes a clustering-based detection method, which can balance the fascicle/aponeurosis tracking accuracy, computational load, and robustness for low-frequency US imaging data. This method aims to mimic a human investigator in labeling the architectural features in an unsupervised learning fashion and thus estimate the PA. This method is derived based on the following facts that fascicles and aponeuroses can be distinguished by clustering the pixels of the US image and they are in tubular shape so that lining the left and right points can be used to determine their orientations.

A human expert likely assigns the tubular shape, that is, cluster, with the highest brightness and length as the targeted muscle fascicles or aponeuroses. Therefore, we can assign values to each cluster based on the length and brightness, and then the cluster with the highest value is selected as the intended muscle fascicle.

The proposed method was tested on the tibialis anterior (TA) muscle US image time sequence data obtained during isometric ankle dorsiflexion experiments from three participants without neuromuscular disorders. One dominant fascicle and the deep aponeurosis in each US image were labeled manually by a human expert. The labeled fascicle’s and aponeurosis’s orientations and the resultant PA measures were treated as the ground truth. We initially investigated the performance of three clustering methods, density-based spatial clustering of applications with noise (DBSCAN) (Ester et al., 1996), K-means (Hartigan and Wong, 1979), and hierarchical agglomerative clustering (HAC) (Lukasová, 1979), which are representatives of density-based, partitioning, and hierarchical clustering, on one out of three human participants. The result indicates that DBSCAN performs superiorly to the others, and the *UltraTrack* outperforms the *ImageJ* on this dataset. Therefore, we compared the result collected from DBSCAN-based clustering and the *UltraTrack*, and the former showed higher accuracy in our low-frequency image data while processing time was approximately 0.2 s per image with an Intel Core i7 Processor. The preliminary results shown in this paper imply that the clustering methods may have value in extracting muscle structural features for their potential use in human–robot interaction control or diagnosing degenerative muscular diseases.

## 2. US Imaging data and ground truth acquisition

Experimental data that were collected in our previous study (Zhang et al., 2020b), including isometric ankle dorsiflexion force at seven ankle joint postures and corresponding TA muscle US imaging signals from three able-bodied participants, were implemented here to test the proposed unsupervised clustering approach in this paper. The detailed experimental setup, US transducer placement, and data collection can be referred to Zhang et al. (2020b). During the experiments, the ankle joint dorsiflexion force signals were collected at 1000 Hz while the B-mode US images of the TA muscle were synchronously collected at a frame rate of 20 Hz. In the experimental data collection procedure, a trigger signal from the data acquisition board (DAQ) was sent to the US machine to synchronize the collection starting time point, and both force and image data were selected every 0.05 s; thus, signals were aligned.

**Figure 1.**
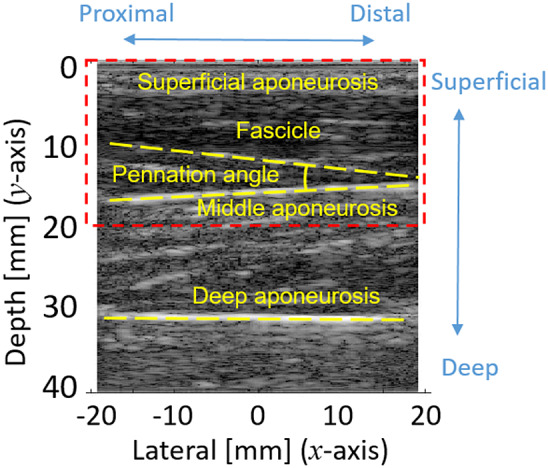
A typical B-mode US image of the TA muscle. 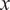 -axis is the distance along the probe’s longitudinal direction (the center line is the zero position, and left and right sides represent proximal and distal directions), and 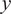 -axis is the depth of the TA muscle. The RGB data of every pixel were transferred to grayscale values between 0 and 255.

A typical B-mode US image of the targeted TA muscle is shown in Figure 1, where the 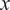 -axis is the distance away from the US transducer center along the longitudinal direction, and the 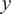 -axis is the depth from the skin surface. The brightness and darkness of the US image represent the normalized gray-scaled value (between 0 and 255) of each pixel, which is calculated from a logarithmically compressed imaging signal. For simplicity, only the upper pennate section is taken into consideration for determining the structural features like fascicle orientation and PA. The upper pennate section is selected as in the red dashed rectangular area in Figure 1, where PA is defined as the angle between the most visualized fascicle and the aponeurosis. The orientation of the fascicle and the middle aponeurosis with respect to the horizontal level is calculated separately to get PA by using the manual label approach and the proposed clustering-based detection approach.

A professionally trained expert labeled the fascicle and aponeurosis in each image as shown in Figure 1. The manual fascicle and aponeurosis selections were based on the brightness, length, position of the former detected fiber, and so forth, which can help design the rules for our unsupervised learning.

## 3. Method

### 3.1. US imaging preparation and pre-processing

The pipeline of muscle fiber detection procedures can be divided into three main phases, including image preparation, clustering and re-clustering, and fiber selection. In this section, the details of the procedures are presented.

#### 3.1.1. Trim

**Figure 2.**
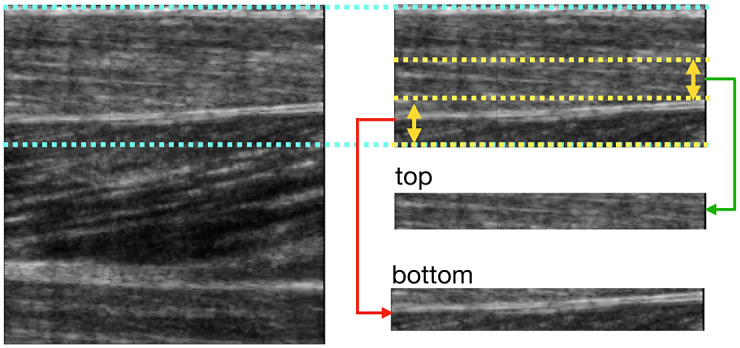
The segmentation of the upper pennate session of TA muscle, where muscle fascicle is in the “top” sub-image, and middle aponeurosis is in the “bottom” sub-image. These two sub-images are the two regions of interest (ROIs) to detect the muscle fascicle and middle aponeurosis, respectively.

As discovered in Figures 1 and 2, only local small regions contain the interested muscle fascicle and middle aponeurosis. In this procedure, we trimmed off the parts from the original image that apparently did not contain the fascicle or aponeurosis. Then, the remaining image was cut into two sub-images, that is, the “top” one, which contains the target fascicle, and the “bottom” one, which contains the middle aponeurosis. The trimmed image can be seen on the right side of Figure 2.

#### 3.1.2. Denoising

In this step, we removed the pixels whose brightness is lower than a predefined threshold, 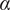 . This can help highlight the muscle fascicles and middle aponeurosis.

#### 3.1.3. Augmentation

For some images where the fascicles were not clear, we augmented the brightness in certain regions. The region is determined based on human judgment. This helped further highlight the target muscle fascicles. The following content demonstrates its effectiveness.

### 3.2. Initial clustering

The clustering technique in this paper groups the total 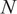 pixels of the ROIs, 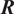 , to 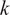 clusters based on the associated parameters, 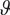 , where 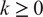 , and the clustering fails if 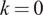 . Considering the case that 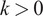 , let’s define the clusters to be 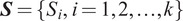 , where 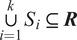 .

#### 3.2.1. DBSCAN

DBSCAN is a density-based clustering method, which has two parameters, that is, the minimum points 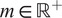 and distance 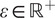 . The clustering rule is that:

1. For one pixel, 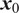 , group all the neighbors, 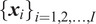 , which are within 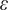 to the pixel. If 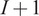 is greater than 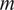 , 
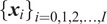
 are defined as the core points.
2. If a pixel, 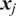 , is within 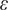 of a core point, 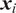 , 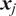 is defined to be directly reachable from 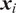 . Assuming that there exists a sequence of core points, 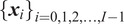 , and 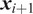 is directly reachable from ***x***_*i*_, ***x***_*j*_ is defined to be reachable from 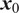 if it is directly reachable from 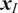 .
3. All the pixels are reachable from a core point and the core point itself forms a cluster.
4. After each pixel is examined, all the clusters are identified. The pixels that do not belong to any cluster are treated as noise.

This clustering mechanism leads to a 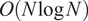 computation complexity in average, and the worst scenario is 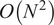 .

#### 3.2.2. K-means

K-means is a partitioning clustering method, which is to find 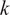 clusters, 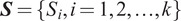 , that can minimize the following within-cluster sum of squares, that is,
(1)

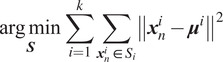

where 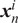 ( 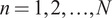 ) denotes the 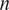 th pixel position in Cluster 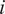 , and 
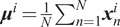
 denotes the mean of Cluster 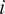 . Note that 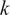 is usually a user-defined constant. The clustering is applied in an iterative manner. Initially, 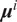 ( 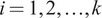 ) is randomly selected and 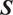 is determined by solving (1). Based on the current 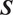 , 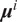 are updated, and thus 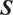 are further updated until converge.

The computation complexity is 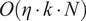 , where 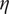 is the number of iterations. In this paper, the dimension of the US image pixel is 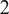 , for example, axial and lateral coordinates in each US image.

#### 3.2.3. HAC

HAC is very similar to K-means, but it does not demand 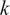 exact clusters and does not randomly pick up 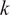 initial means. Instead, each pixel is initially treated as a cluster, and each of the neighboring pixels of the cluster is absorbed into a cluster if the distance, Euclidean distance, is less than a user-defined threshold. Typically, the computation complexity of the naive HAC is 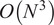 .

### 3.3. Re-clustering

After the pixels were grouped into 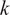 clusters, we performed a re-clustering process to drive them to 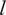 new clusters. The purpose of this process was to update 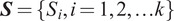 to 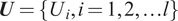 , where 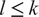 . The procedures are summarized as the following two steps.

**Figure 3.**
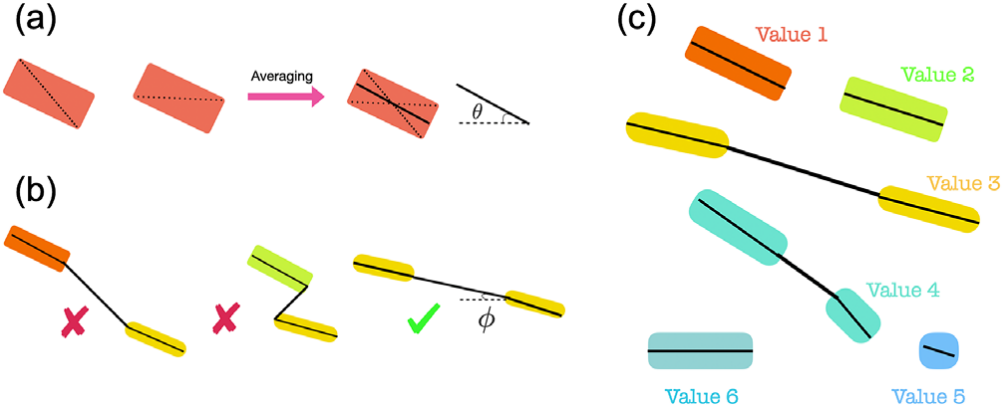
(a) The definition of the tubular shape muscle fascicle orientation angle through clustering, (b) the criteria of reclustering to merge two clusters into one, and (c) each cluster is assigned a value.

Step 1. For each cluster, find the pixel points in the four corners, that is, right-up, 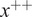 , right-down, 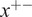 , left-up, 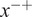 , and left-down, 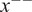 . Draw the first line, 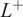 , that aligns point 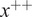 and point 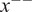 , and the second line, 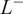 , that aligns point 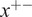 and point 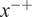 . Denote the intersection angle of 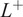 and horizontal direction to be 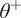 , also known as the orientation angle of 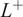 . Similarly, the orientation angle of 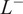 is denoted as 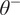 . Then, compute the average, 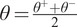 , which is defined as cluster angle (the definition is visualized in Figure 3(a)). As the cluster has a tubular shape, the angle 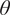 could roughly represent the orientation.

Step 2. As shown in Figure 3(b), we find each pair of clusters, if one’s most left point is on the right of the most right point of the other’s, which have similar orientation angles, that is, 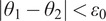 . Then, the connect these two clusters by lining the point 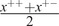 of the left cluster and 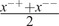 of the right cluster. Defined the angle between the connection line and the horizontal direction as connection angle, 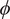 . Then, check if the values of 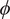 and 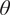 are very close, that is, 
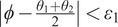
 . If so, the clusters are regrouped into one (see Figure 3(b)). Iterate this procedure until no clusters can be merged. We can remove the implausible clusters by applying domain knowledge of the general fascicles flow in the US image. For instance, the fascicle’s most left pixel position should be higher than the most right one; otherwise, the cluster is not plausible and, therefore, it is discarded.

Then, the orientation of the newly merged clusters, defined as 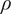 (computed as shown in Figure 3(a)), is used to represent the orientation of the interested fascicle or aponeurosis.

### 3.4. Target muscle selection

In this procedure, we assigned a value to each cluster using a user-defined function, and then selected the cluster with the highest value as the targeted muscle, for example, fascicle. The value function of 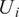 (the 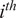 cluster) is defined as
(2)

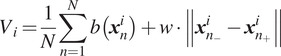

where 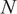 is the pixel number in that cluster, 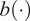 is the echo intensity of a pixel, 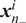 is the position of the most left pixel, 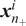 is the most right pixel in 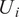 , and 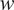 is the weight. The objective was to find the cluster that carried the highest value, that is, 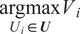 . Then the angle between the line that connects 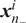 and 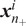 with respect to the horizontal direction was defined as the orientation angle of the new cluster, which was also considered as the orientation of the targeted muscle fascicle. By summing the two orientation angles of muscle fascicle and middle aponeurosis, we obtained the PA for the TA muscle.

## 4. Analysis of representative samples

### 4.1. A representative image

**Figure 4.**
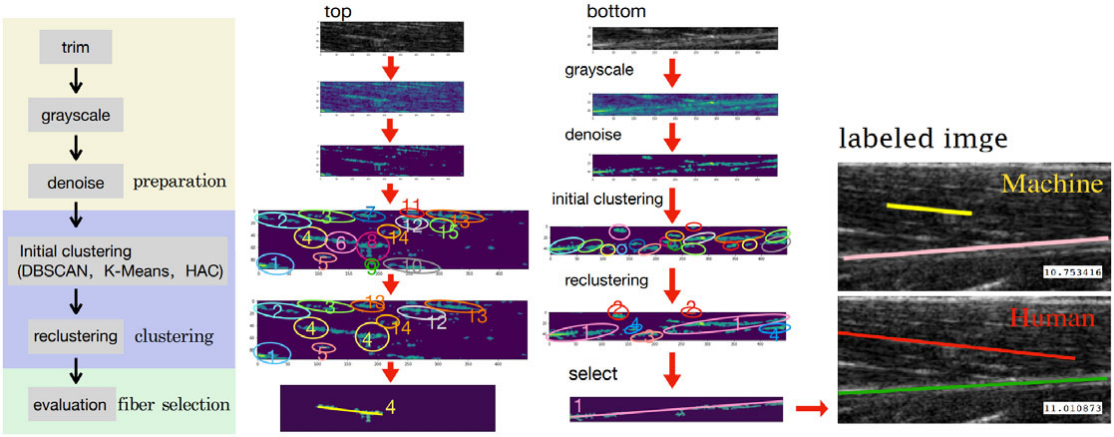
The pipeline of the muscle fascicle detection and the details of the image processing for each step. The yellow line in the machine-labeled image represents the orientation of the cluster with the highest value in the top image, and the purple one is for the down image.

We tested the pipeline step by step shown in Figure 4 on a representative image. After the denoise operation, the sub-images contained much fewer pixels than the original ones. Then the clustering can be effectively applied to the denoised sub-images as the tubular shapes in the image became clearer. From the second and third columns in Figure 4, we can see that some tubular segments were grouped into several clusters. In this representative image, the pixels in the top sub-image are initially clustered into 15 clusters. After the re-clustering procedure, the clusters in the top sub-image were updated to eight new clusters. Without losing generality, the fascicle detection results will be highlighted in the following content. After evaluating the new clusters, the one with the highest evaluation value was found, and a line that connects its most left-up pixel point and most right-down pixel point was drawn to represent the muscle fascicle.

### 4.2. Augmentation filter

Although the fascicle detection performance is satisfactory for the representative image with less noise, there is a possibility for hindered performance with blur images containing significant noise.

**Figure 5.**
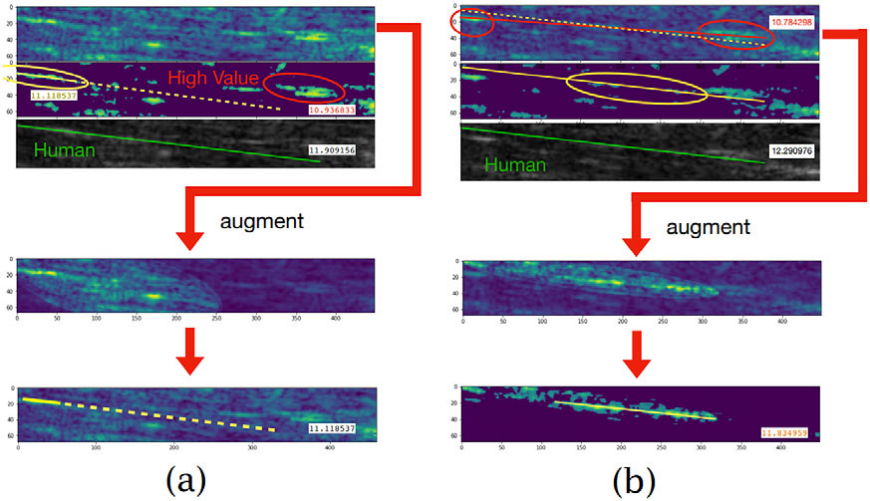
Showing how the augmentation filter improves the detection accuracy in the presence of (a) a high-value noise cluster and (b) an unclear cluster.

From Figure 5(a), we can see a cluster that does not contain a fascicle but carries a very high value as its brightness is high. Its high value is very likely to induce a wrong detection in the initial clustering. We can see that if the detection is attracted by the wrong muscle, which is circled by a red ellipse, the PA is estimated to be 10.93°. If right, the PA is 11.11°, while the human-annotated PA is 11.90°. To address this problem, we augmented a local region in the top sub-image that contained the targeted fascicle. This operation aims to increase the brightness of the pixels in that region so that the fascicle is highlighted. In this study, we designed an ellipse augmentation region as shown in Figure 5. The focus and vertex of the ellipse are user-defined. In practice, we usually only need to augment the top sub-image containing muscle fascicles because the bottom sub-image containing middle aponeurosis is often sufficiently clear. From Figure 5, we can see that the accuracy of the detection is ensured after applying the augmentation filter.

Except for the disturbance with higher brightness that may affect the initial clustering, another case may also cause the wrong initial clustering. As shown in Figure 5(b), the fascicle is not clearly shown. This is probably because this muscle fascicle is shadowed by the fat or connective tissues. From the results in Figure 5(b), we can see that after the augmentation is applied the muscle fascicle detection succeeds.

We can consider the augmentation filter as an assistant and optional procedure. When there are some sliding movements between the US transducer and the skin, the denoising procedure could eliminate undesired noise to make the blurred image clearer. However, extremely low and high denoising is likely to fail the detection task since the filter may either remove useful information or not denoise ineffectively. In that case, an augmentation filter can also be applied to enhance the local region containing the targeted fascicle to address the problem.

### 4.3. Viscosity

For some other situations, the fascicle barely showed in the image, so it could not be detected no matter how the augmentation was designed. In this case, the investigator would guess the location of the muscle fascicle based on the previous image. If the loss of detection is only occasional, the tracking was assumed valid. To mimic this capability of the investigator, we designed a viscosity function, which is defined as. 
(3)

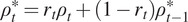

where 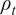 is the measured angle at time 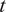 , 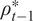 and 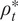 are the determined angles at 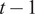 and 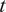 , respectively, and 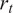 is the weight that is defined as
(4)

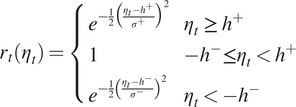

where 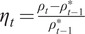 and 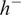 , 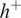 , 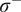 , and 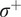 are parameters. Therefore, 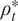 is used as the final angle. This method means that if the fascicle is not shown properly, the detected PA is abnormal, and thus, the weight is nearly 0. In this case, the previously-detected PA will be assigned to the final PA. If the PA is normal, the weight of the current detection dominates.

**Figure 6.**
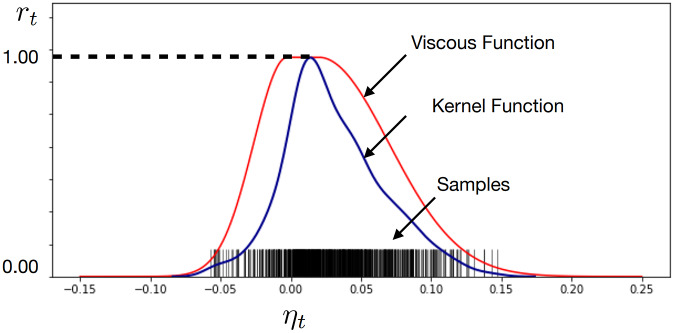
The designed viscous function. The vertical bars are samples, that is, the real incremental data. The Kernel function is the Kernel Density Estimation upon the samples using scikit-learn:

We can see that (4) is a skew-Gaussian type distribution function. In this paper, we determined the parameters depending on the real US imaging data collected from two able-bodied subjects (i.e., A02 and A03 that will be shown later). In the dataset, we designed a value 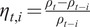 , where 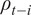 is the human determined angle at 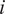 steps/frames ahead. In this case, the 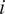 was iterated from 1 to 10. The data distribution, its corresponding kernel function, and our viscosity function are shown in Figure 6. The parameters, 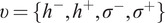 , for the viscosity function have to be selected such that the span width of the viscosity function is wider than that of the kernel function. Therefore, the viscosity function would only eliminate the outliers but not the normal data.

### 4.4. Fine-tuned results

**Figure 7.**
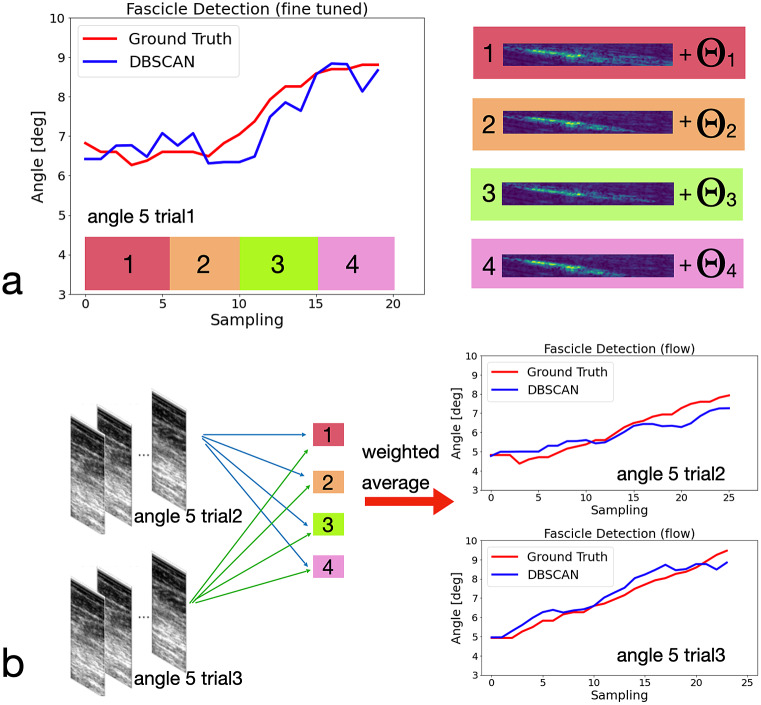
The accuracy of (a) the detection of the fascicle orientation with fine-tuned parameters using the DBSCAN clustering method and (b) the image flow with the viscosity function and the fine-tuned parameters. In (a), four sets of parameters were used individually on different frames to obtain accurate estimations. In (b), the four sets of parameters were used simultaneously on all frames, and the weighted average was taken to give the final estimation of the muscle orientations.

We selected the collected data from a trial when the ankle joint posture was set at 5° dorsiflexion (the 0° posture means that the shank is perpendicular to the sole of the foot), and the US imaging data contained 20 images. We elaborately designated the parameters, 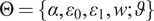 , and the shape of the augmentation filter for each image. We found four types of augmentation filters and parameter sets that gave a satisfactory result for fascicle detection. The first one was used for images 1–6, the second was for 7–9, the third one was for 10–15, and the fourth one was for 16–20. The performance is shown in Figure 7(a).

It is worth noting that the augmentation filter may vary across different images. Therefore, to facilitate automation, we applied the viscous function again to compute the weighted average, 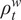 ,
(5)

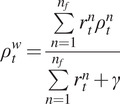

where 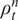 is the angle detected using the n-th filter, 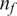 is the number of the filters, 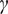 is a small value that prevents the calculation result in (5) from blowing up, 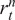 is the weight computed using (4) and set 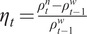 . If an augmentation filter is suitable for the image we have 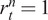 . If it is not, 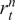 will be very small so that this angle is rolled out. We applied this method to the two other trials for this subject with 5° dorsiflexion in flow (time sequence), and the results can be seen in Figure 7(b).

### 4.5. US image time sequence tracking

**Figure 8.**
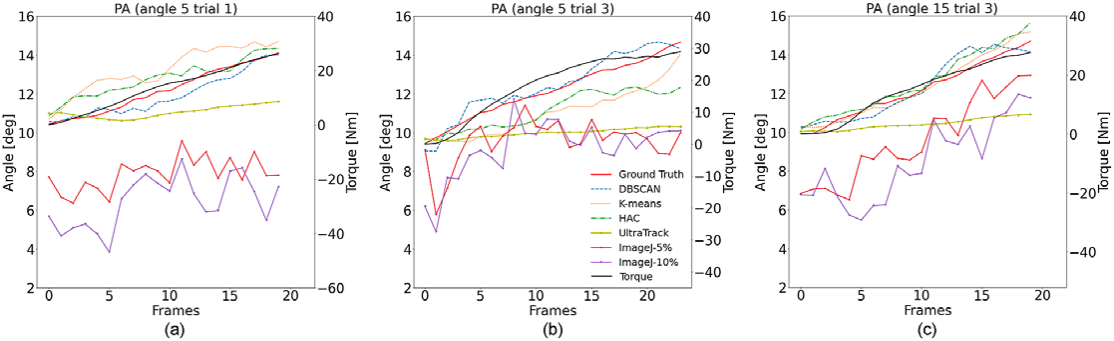
The PA detection using our method with DBSCAN, K-means, and HAC clustering, and the benchmark methods, that is, *UltraTrack* and *ImageJ.* The data shown here are from the first participant at dorsiflexion angles 5° (Trial 1 and 3) and 15° (Trial 3). Also, the isometric ankle torques were plotted to show a high correlation with the PA sequences.

Finally, we also investigated the automatic muscle fascicle detection based on our newly-derived pipeline with DBSCAN clustering. The promising results can guide us to apply the method to estimate PA using all clustering methods (DBSCAN, K-means, and HAC). The pseudo-code of the algorithm is shown in Table 1 for a clear presentation. Results from three representative trials when the ankle joint posture was set at 5° and 15° dorsiflexion are shown in Figure 8. Furthermore, to quantitatively evaluate the PA tracking performance with the proposed method, the root-mean-square error (RMSE) values between the detection by using the proposed method and the human expert labeling from nine repeated trials when the ankle joint posture was set as 5°, 10°, and 15° dorsiflexion are summarized in Table 2.

**Table 1.** Showing the algorithm flow of how to detect the PA of the data stream

	Algorithm (pseudo code)
1:	Set the parameters 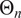 , 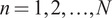
2:	Measure 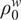 manually
3:	Select the clustering method (DBSCAN, K-means, or HAC)
4:	Set augmentation ellipse region (optional).
5:	*For* each image, 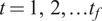 , in the trial:
6:	Set 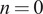 , 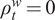
7:	*For* 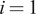 to 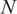 :
8:	trim the image to top and bottom sub-images
9:	denoise both sub-images
10:	do clustering to obtain 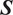 for both sub-images
11:	update 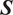 to 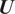
12:	assign value 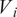 to each 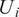 in 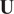
13:	solve 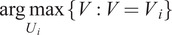 to select the cluster 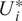
14:	compute PA 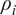 from 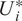 for top and bottom sub-images
15:	compute 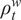 using (5)
16:	compute 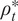 using (3)
17:	*End*
18:	*End*

**Table 2.** Summary of PA detection results by using the proposed approach on one representative participant’s TA muscle during the ankle joint at different postures

Results of the PA detection							
	RMSE [°]						
Angle 5°	DBSCAN	K-means	HAC	*UltraTrack*	*ImageJ*-5%	*ImageJ*-10%	Data Siz**e**
Trial 1	0.419	1.140	0.723	1.939	4.714	6.169	20
Trial 2	0.539	1.171	0.374	2.170	3.373	4.771	26
Trial 3	0.484	1.220	1.210	2.525	3.795	4.138	24
Angle 10°							
Trial 1	0.358	0.357	0.994	1.698	2.424	3.848	15
Trial 2	0.892	1.143	0.980	0.934	2.817	3.863	25
Trial 3	1.053	0.632	0.539	1.649	2.787	3.357	23
Angle 15°							
Trial 1	0.647	1.134	1.800	1.892	4.077	4.278	20
Trial 2	1.118	0.489	0.538	2.729	3.848	6.327	19
Trial 3	0.523	0.300	0.494	2.115	4.553	5.671	20
Overall	0.727	0.948	0.941	1.951	3.598	4.713	192

Abbreviations: DBSCAN, density-based spatial clustering of applications with noise; HAC, hierarchical agglomerative clustering; PA, pennation angle; RMSE, root mean square error; TA, tibialis anterior.

### 4.6. Comparison with existing algorithms

The detected PA results by using the newly-proposed clustering method were compared with mature benchmark techniques, for example, *UltraTrack* and *ImageJ.*

#### 4.6.1. UltraTrack

**Figure 9.**
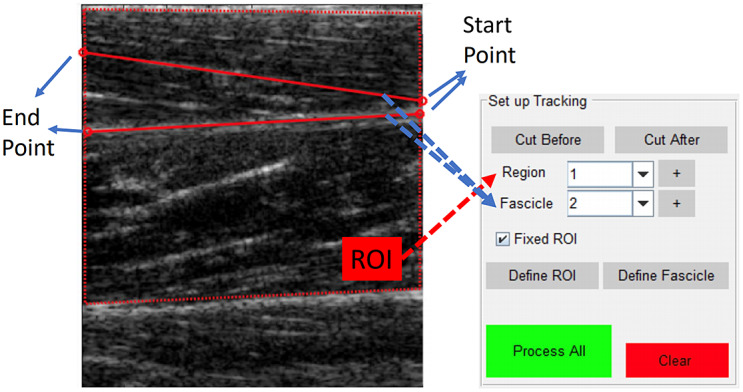
The control panel and operation demonstration of using *UltraTrack* (optical flow).

As introduced before, *UltraTrack* detects objects by tracking key points of figures. The operation of using the Matlab toolbox is to upload the video stream of B-mode US images first, and then, create ROI and select the starting/ending points for each interested muscle fascicle in the first image frame, and then, start processing. The graphical user interface when using *UltraTrack* is shown in Figure 9. In our case, one ROI, one superficial muscle fascicle (upper red line), and the middle aponeurosis (lower red line) were selected in the initial image frame of each trial. The tracked PA values from the US image time sequence were used for comparison with the PA values by using the proposed clustering approach. In addition, the RMSE values between the *UltraTrack*-derived PA and human expert-labeled PA were also calculated to evaluate the detection performance, as reported in Table 2.

#### 4.6.2. ImageJ

**Figure 10.**
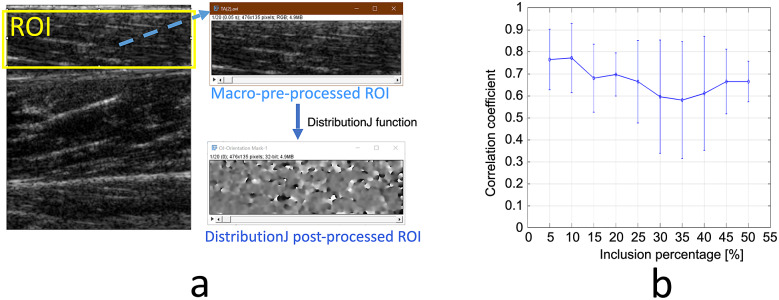
(a) The highlighted ROI (the ROI selected for cropping of the TA muscle fascicles), macro-pre-processing ROI (the ROI in the image after the image went through the functions within the macro), and post DistributionJ processing of an US image frame (the ROI after the DistributionJ function that can be used to determine the orientation distribution). (b) The mean and standard deviation of the correlation coefficient between the *ImageJ* detection angle and the ground truth across different inclusion percentages.

B-mode US image videos from the TA muscle during isometric ankle dorsiflexion movement were also processed utilizing *ImageJ* and statistically analyzed via Matlab. *ImageJ*’ s open-source image processing package, Fiji (Schindelin et al., 2012), was used to analyze the orientation distribution of the middle aponeurosis-like segments and muscle fascicle-like segments from both the lower half and upper half of the TA muscle. Each image was cropped based on the ROI with the rectangle area section tool to remove external information such as image edges and unrelated fascicles (Figure 10(a)). According to procedures in Seynnes and Cronin (2020), a custom macro that ran a series of functions was followed to improve image clarity for better fascicle definition. The *Subtract Background* function was run to remove large variations in the background intensities of the video. The *Non-Local-Means-Denoising* and median filter was applied to remove extraneous noise, and then the video was converted to 32 bits. The *DistributionJ* within the *OrientationJ* plug-in was then utilized to create a table with the distribution of the orientation angle of the targeted aponeurosis and muscle fascicle. The TA muscle’s PA was calculated and statistically analyzed via MATLAB. The PA value was calculated by taking the weighted average from the top 5% to 50% (with an increment of 5%, defined as inclusion percentage) of the selected pixel clouds that represent the middle aponeurosis and the TA muscle fascicle. The sum of the weighted averages for the varying percentages of the middle aponeurosis and TA muscle fascicle were then totaled to obtain the PA value of different weighted averages. The correlation coefficient between the *ImageJ*-derived PA and the human expert label was calculated for each weighted average. The quantitative results can be seen in Figure 10(b), where correlation coefficients are relatively higher when selecting the top 5% and 10% of the pixel clouds for the weighted average PA calculation. Therefore, we selected the top 5% and 10% as the representatives for the comparison. The RMSE values between the *ImageJ*-derived PA and human expert-labeled PA are reported in Table 2.

## 5. Result

From those PA tracking comparison results along the US image time sequence, we can observe that the proposed method with DSCAN as the initial clustering method outperforms other algorithms and the *UltraTrack* outperforms *ImageJ* in our dataset. Quantitative results of the PA tracking RMSE between the human expert labeling and the detection with different methods from multiple trials and multiple ankle joint postures can be seen in Table 2. Therefore, we would select the proposed DSCAN-embedded two-step clustering approach and the *UltraTrack* toolbox for further comparison testing.

Then, we tested our proposed method with DBSCAN and the *UltraTrack* on three able-bodied participants, that is, A01, A02, and A03. Each subject’s ankle joint initial dorsiflexion posture was set at three different postures 5°, 10°, and 15°, respectively. We tested three trials under each posture and plotted the error between the detected PA and the human expert-labeled ground truth in Figure 12.

From the figures, we can see that the mean error values for the three subjects using the proposed method are 0.03°, −0.55°, and − 0.63°, respectively, while the standard deviation (the red lines) are 0.70°, 0.94°, and 0.96°, respectively. The mean error using the *UltraTrack* are 0.41°, −1.40°, and − 0.76°, respectively, while the standard deviation (the red lines) are 1.10°, 1.38°, and 0.97°, respectively. The figure shows that the proposed method slightly outperforms the *UltraTrack* method in our dataset.

## 6. Discussion and conclusion

In this paper, we developed a clustering-based detection method, which mimics a human investigator in terms of labeling the muscle fascicle on US images in an unsupervised learning fashion. In this preliminary study, we focused on detecting the orientation of the TA muscle’s fascicle and aponeurosis, and thus calculating PA automatically.

The results collected by the proposed method were compared with those obtained by applying benchmark techniques: the optical flow in *UltraTrack* and *ImageJ.* The performance of the proposed clustering methods in our dataset is shown more precise in RMSE when compared to the benchmark methods.

**Figure 11.**
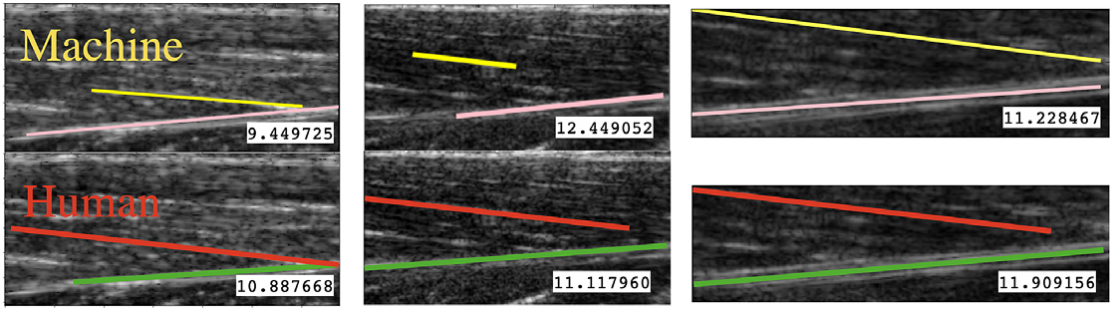
Some representative tracking results. It also shows that even if the fascicles are correctly detected, there is still a considerable error in the PA calculation.

Although the preliminary study shows promising results for potential real-time implementation, there are still some limitations given that the method is still at a rudimentary phase. First, both *UltraTrack* and *ImageJ* can also measure the length of fascicle/aponeurosis, while our proposed method can only measure the muscle fascicle/aponeurosis orientation. Also, the orientation detection is very sensitive. For example, in the case shown in Figure 11, even if the fascicle is correctly detected, the calculation of PA may still have a non-neglectable error due to the high sensitivity of the muscle orientation. This can perhaps explain why the PA tracking result with the proposed method does not look as smooth as the tracking results with the *UltraTrack* method. In fact, the smoothness of the tracking result is an important index because the PA is likely to be used for human joint movement intention prediction in a continuous manner, which is an innovative and noninvasive approach for human–machine interface for implementing rehabilitative and assistive devices. If the PA tracking smoothness is weak, the continuity of human joint movement intention prediction could be affected, thus deteriorating the effective performance of those devices. The method is sensitive to parameter selection, including denoising factors, augmentation filters, viscous functions, and so forth. In this work, we tried our best to select parameters for the proposed and benchmark methods to deliver the best performance. But, arbitrary parameters may lead to unsatisfactory results. Finally, the processing time is still too long to be implemented in real time in terms of a feedback control system with higher control frequency. The US images were collected at 20 frames per second, but the processing time for each image with DBSCAN, K-means, and HAC methods can take approximately 0.2–0.4 s per image with an Intel Core i7 Processor (CPU). Potentially, the processing time will be further reduced by applying a graphical processing unit (GPU). Nevertheless, the complexity of the proposed method is already low compared to the existing methods mentioned in the literature. For example, BLST is already a method with the lowest complexity, 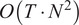 , among all the aforementioned existing methods, where the variable 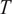 denotes the number of rotations that is usually comparable to the image size 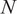 . Therefore, we can roughly estimate the complexity of BLST to be 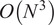 , which is equivalent to HAC, that is, the clustering method with the highest complexity among the methods we investigated. There is also space for improvement in the experiment design. In this paper, the image data was collected using isometric contraction, which may cause muscle distortion. In future work, fixed-end contraction will be adopted (Raiteri and Hahn, 2019). In future work, we will also take the fascicle length into consideration. It requires an algorithm that can precisely detect the boundary of the muscle fiber, especially the start and end. The missing part of the muscle can be extrapolated, and the PA estimated by the methods presented in this paper may help with this (Franchi et al., 2020).

**Figure 12.**
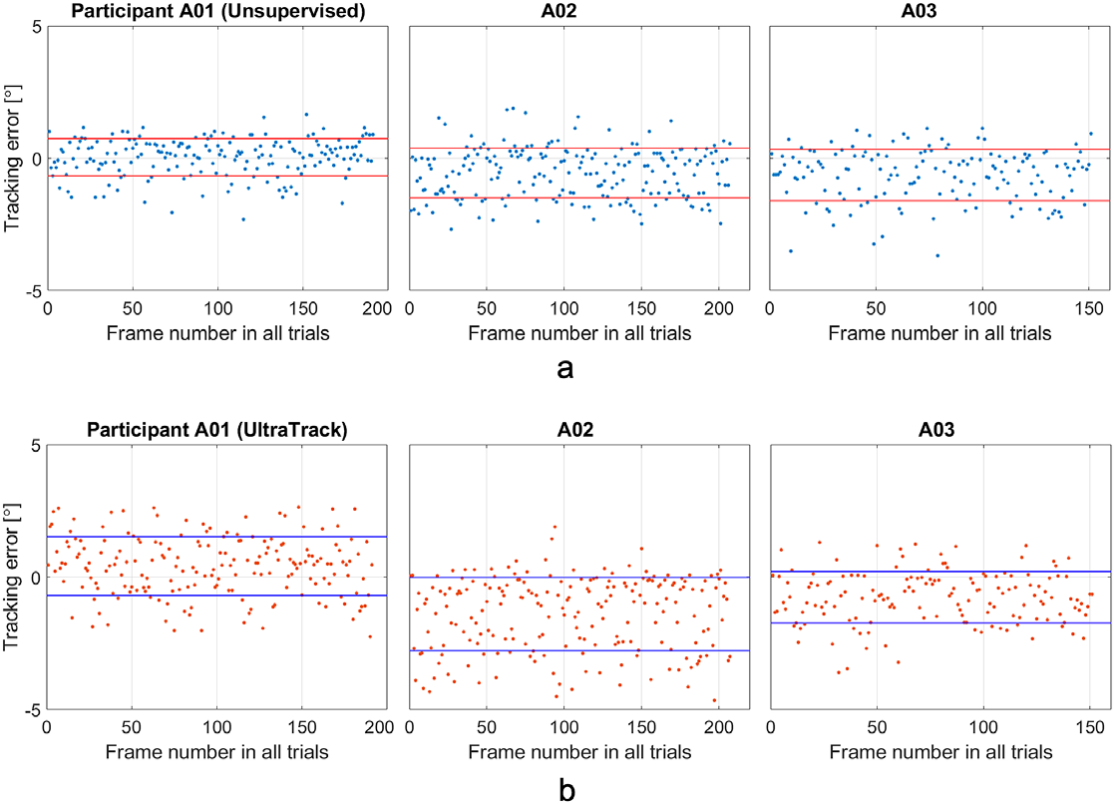
(a) The tracking error of the proposed method, and (b) the *UltraTrack.* In (a), the mean error for the three subjects are 0.03°, −0.55°, and −0.63°, respectively, while the standard deviation (the lines) are 0.70°, 0.94°, and 0.96°, respectively. In (b), the mean error for the three subjects are 0.41°, −1.40°, and −0.76°, respectively, while the standard deviation (the lines) are 1.10°, 1.38°, and 0.97°, respectively.

In conclusion, the proposed clustering-based detection method estimates the PA using US images with high precision in our dataset. Additionally, it demonstrates that this method can mimic a human investigator in terms of labeling the skeletal muscle’s fascicle and aponeurosis. Because of the various merits (e.g., intelligence, robustness, and flexibility) that human investigators possess, the methods aiming to emulate expertise in inferring US images will be explored further in the future.

## Supplementary material

For supplementary material accompanying this paper visit https://doi.org/10.1017/wtc.2022.30. click here to view supplementary material
