# Supplementary material for: A clustering-based method for estimating pennation angle from B-mode ultrasound images
Source: Wearable Technol. 2023 Mar 1;4:e6. doi: 10.1017/wtc.2022.30 (PMC10936288; doi:10.1017/wtc.2022.30)
Supplement: Supplementary file 1 [file S2631717622000305sup001.pdf]

Supplementary Material

In this supplementary material, we will show the codes used to write the paper. We can directly run the main function in the terminal. After open the terminal, we should find the correct path to find the main function (i.e., root folder). Then we just need to type 'python main'. One example is shown below.

```
Last login: Sun Jan 29 14:18:10 on ttys000

The default interactive shell is now zsh.
To update your account to use zsh, please run `chsh -s /bin/zsh`.
For more details, please visit https://support.apple.com/kb/HT208050.
(base) Xs-MacBook-Pro:~ xbao$ cd documents
(base) Xs-MacBook-Pro:documents xbao$ cd codes
(base) Xs-MacBook-Pro:codes xbao$ cd cv
(base) Xs-MacBook-Pro:cv xbao$ cd integrate3.1
(base) Xs-MacBook-Pro:integrate3.1 xbao$ python main
----------------------
Image NO.:  1
filter0 worked
filter1 worked
filter2 worked
filter3 worked
filter4 worked
filter5 worked
filter6 worked
filter7 worked
final_angle: 12.595013
Running Time 3.716571807861328



----------------------
----------------------
Image NO.:  2
filter0 worked
filter1 worked
filter2 worked
filter3 worked
filter4 worked
filter5 worked
filter6 worked
filter7 worked
final_angle: 12.881586
Running Time 3.694883108139038



----------------------
----------------------
Image NO.:  3
filter0 worked
filter1 has compilling error
filter2 worked
filter3 has compilling error
filter4 worked
filter5 worked
filter6 worked
filter7 worked
final_angle: 13.248520
Running Time 3.71648907661438



----------------------
----------------------
Image NO.:  4
filter0 worked
```

Note: The labeled filters (i.e., sets of parameters) fail to deliver valid detection. Then, they will be rolled out from the final result (i.e., final angle). Actually, this is common. We do not expect all filters to work. Also, the machine we are using to show the demo is slower than when we were writing the paper. Therefore, the Running Time is usually above 3.5s, and they were below 3s before. The details of the codes are shown in the following contents.

```
#import standard modules

import cv2
import time
import numpy as np
#from numpy import *
import pandas as pd
#from pylab import *
import math

#import my modules
import Fiber_Select as fs


#default parameters
b1 = 80 #delete the pixels whos brightness are lower than b1
b2 = 90 #delete the pixels whos brightness are lower than b2

connectivity1 = 4 #connectivity for cv2.connectedComponentsWithStats
for image1
connectivity2 = 4 #connectivity for cv2.connectedComponentsWithStats
for image2

n_clusters1 = 40 #number of cluster to be grouped for image1
n_clusters2 = 10 #number of cluster to be grouped for image2

eps1 = 4.5
eps2 = 5.5
mini1 = 20
mini2 = 50

tol_deg_in_cluster1 = 3 #degree for checking if two clusters are
paralell for image1
tol_deg_out_cluster1 = 3 #degree for checking if two clusters are
alined for image1
tol_deg_in_cluster2 = 5 #degree for checking if two clusters are
paralell for image2
tol_deg_out_cluster2 = 5 #degree for checking if two clusters are
alined for image2

w1_1 = 0.2 #weight on total brightness for image1
w2_1 = 50 #weight on fiber length for image1

w1_2 = 0.2 #weight on total brightness for image2
w2_2 = 200 #weight on fiber length for image2


aug_visc1 = []
aug_visc2 = []
aug_visc = []
```

```
time_visc = []

sigma1 = math.sqrt(2)
sigma2 = math.sqrt(2)

limit1 = 25
limit2 = 100

case_number = 7

angle_weighted1 = 0
angle_weighted2 = 0
angle_weighted_sum1 = 0
angle_weighted_sum2 = 0

w1 = 0
w2 = 0


#--------------------Select Folder Here-------------------
case = 'Ankle_A01/angle_15_trial3'
image_path = './data/' + case + '/Original/TA'

num_total = 20
start_id = 54


last_angle1 = 6.2
last_angle2 = 3.6



last_angle = last_angle1 + last_angle2




#trim the images
left_most = 220    #3
right_most = 690   #4
up_most = 95      #1
down_most = 580    #2

trim_ver_start = 40
trim_ver = 190
trim_hor = 450
basic_cut = 80
```

```
zoom_end1 = 45
zoom_end2 = 115
zoom_end3 = 350
zoom_end4 = 450
zoom_bright = 85




#-----------------------------------------------------------
#start



for i in range(num_total):
    print('---------------------')
    print('Image NO.: % 2d' %(i + 1))

    start_time = time.time()


    image = cv2.imread(image_path + str(i + start_id) + '.tif',
cv2.IMREAD_GRAYSCALE)

    image_o_t = fs.trim_image(image, left_most, right_most, down_most,
up_most)


    #find the cutting position
    image_t_t= image_o_t.astype(np.uint8)
    image_zoom = image_t_t[zoom_end1:zoom_end2, zoom_end3:zoom_end4]
    threshold = np.percentile(image_zoom, zoom_bright)
    image_zoom_clean = fs.denoising(image_zoom, threshold)
    image_zoom_clean = np.array(image_zoom_clean, dtype=np.uint8)
    cut_position = fs.find_cut_position(image_zoom_clean)

    #cut the image
    image1, image2 = fs.cut_image_general(image_o_t, trim_ver,
trim_ver_start, trim_hor, basic_cut, cut_position)


    for n in range(case_number+1):

        if n == 0:
            b1 = 90
            b2 = 90
            eps1 = 4.5
            eps2 = 5.5
            mini1 = 20
            mini2 = 50
```

```
            tol_deg_in_cluster1 = 3
            tol_deg_out_cluster1 = 3
            tol_deg_in_cluster2 = 5
            tol_deg_out_cluster2 = 5

            w1_1 = 0.2
            w2_1 = 50

            w1_2 = 0.2
            w2_2 = 200
            limit1 = 25
            limit2 = 100
            #augment the image

            #0
            right_pixel1_last, left_pixel1_last, right_pixel2_last,
left_pixel2_last \
            = np.array([71, 461]), np.array([11, 1]), np.array([41,
-11]), np.array([-11, 509])

            image_augment1 = fs.ellipse_augmentation7(image1,
right_pixel1_last, left_pixel1_last, \
                                                       0.00250, 0.0075,
0.00925, 3.75, 3.25, 2.00)

            image_augment2 = fs.ellipse_augmentation7(image2,
right_pixel2_last, left_pixel2_last, \
                                                       0.000550, 0.001,
0.0075, 2.75, 2.5, 1.75)




        elif n == 1:
            b1 = 80
            b2 = 80
            eps1 = 4.5
            eps2 = 5.5
            mini1 = 40
            mini2 = 10
            tol_deg_in_cluster1 = 2.75
            tol_deg_out_cluster1 = 2.75
            tol_deg_in_cluster2 = 5
            tol_deg_out_cluster2 = 5
            w1_1 = 0.2
            w1_2 = 0.2
            w2_1 = 10
            limit1 = 30
```

```
            limit2 = 40
            w2_2 = 200


            #1

            right_pixel1_last, left_pixel1_last, right_pixel2_last,
left_pixel2_last \
            = np.array([65, 390]), np.array([11, 1]), np.array([65,
1]), np.array([20, 509])

            image_augment1 = fs.ellipse_augmentation7(image1,
right_pixel1_last, left_pixel1_last, \
                                                    0.00250, 0.0075,
0.00925, 3.75, 3.25, 2.00)

            image_augment2 = fs.ellipse_augmentation7(image2,
right_pixel2_last, left_pixel2_last, \
                                                    0.000550, 0.001,
0.0075, 2.75, 2.5, 1.75)




        elif n == 2:
            b1 = 75
            b2 = 95
            eps1 = 4.5
            eps2 = 5.5
            mini1 = 20
            mini2 = 40

            tol_deg_in_cluster1 = 3
            tol_deg_out_cluster1 = 3
            tol_deg_in_cluster2 = 5
            tol_deg_out_cluster2 = 5

            w1_1 = 0.2
            w2_1 = 50

            w1_2 = 0.2
            w2_2 = 200
            limit1 = 25
            limit2 = 100
            #2
            right_pixel1_last, left_pixel1_last, right_pixel2_last,
left_pixel2_last \
            = np.array([61, 350]), np.array([13, 1]), np.array([65,
1]), np.array([14, 511])
```

```
            image_augment1 = fs.ellipse_augmentation7(image1,
right_pixel1_last, left_pixel1_last, \
                                                    0.00050, 0.001,
0.0025, 3.75, 3.25, 2.00)

            image_augment2 = fs.ellipse_augmentation7(image2,
right_pixel2_last, left_pixel2_last, \
                                                    0.000550, 0.001,
0.0075, 2.75, 2.5, 1.75)


        elif n == 3:
            b1 = 70
            b2 = 90
            limit1 = 30
            limit2 = 45
            eps1 = 4.5
            eps2 = 5.5
            mini1 = 20
            mini2 = 50
            tol_deg_in_cluster1 = 3
            tol_deg_out_cluster1 = 3
            tol_deg_in_cluster2 = 5
            tol_deg_out_cluster2 = 5
            w1_1 = 0.2
            w2_1 = 50
            w1_2 = 0.2
            w2_2 = 200

            #3
            right_pixel1_last, left_pixel1_last, right_pixel2_last,
left_pixel2_last \
            = np.array([65, 390]), np.array([11, 1]), np.array([65,
1]), np.array([16, 509])

            image_augment1 = fs.ellipse_augmentation7(image1,
right_pixel1_last, left_pixel1_last, \
                                                    0.00250, 0.0075,
0.00925, 3.75, 3.25, 2.00)

            image_augment2 = fs.ellipse_augmentation7(image2,
right_pixel2_last, left_pixel2_last, \
                                                    0.000550, 0.001,
0.0075, 2.75, 2.5, 1.75)

        elif n == 4:
            b1 = 70
            b2 = 90
            eps1 = 3.5
            eps2 = 5.0
```

```
            mini1 = 20
            mini2 = 10
            tol_deg_in_cluster1 = 3
            tol_deg_out_cluster1 = 3
            tol_deg_in_cluster2 = 5
            tol_deg_out_cluster2 = 5
            w1_1 = 0.2
            w2_1 = 50
            w1_2 = 0.2
            w2_2 = 200
            limit1 = 40
            limit2 = 100

            #4
            right_pixel1_last, left_pixel1_last, right_pixel2_last,
left_pixel2_last \
            = np.array([65, 350]), np.array([11, 1]), np.array([65,
1]), np.array([16, 511])

            image_augment1 = fs.ellipse_augmentation7(image1,
right_pixel1_last, left_pixel1_last, \
                                                       0.00575, 0.00995,
0.015725, 3.75, 3.25, 2.00)

            image_augment2 = fs.ellipse_augmentation7(image2,
right_pixel2_last, left_pixel2_last, \
                                                       0.0020, 0.005,
0.0075, 2.75, 2.5, 1.75)

        elif n == 5:
            b1 = 60
            b2 = 90
            eps1 = 4.5
            eps2 = 5.5
            mini1 = 20
            mini2 = 10
            tol_deg_in_cluster1 = 3
            tol_deg_out_cluster1 = 3
            tol_deg_in_cluster2 = 5
            tol_deg_out_cluster2 = 5
            w1_1 = 0.2
            w2_1 = 50
            w1_2 = 0.2
            w2_2 = 200
            limit1 = 40
            limit2 = 100

            #4
            right_pixel1_last, left_pixel1_last, right_pixel2_last,
left_pixel2_last \
```

```
            = np.array([65, 350]), np.array([11, 11]), np.array([65,
1]), np.array([16, 511])

            image_augment1 = fs.ellipse_augmentation7(image1,
right_pixel1_last, left_pixel1_last, \
                                                  0.00050, 0.001,
0.0025, 3.75, 3.25, 2.00)

            image_augment2 = fs.ellipse_augmentation7(image2,
right_pixel2_last, left_pixel2_last, \
                                                  0.000550, 0.001,
0.0075, 2.75, 2.5, 1.75)


        else:
            b1 = 90
            b2 = 80
            image_augment1 = image1
            image_augment2 = image2


        #there are two methods can be used ---- 'KMeans',
'SpectralClustering', 'AgglomerativeClustering', 'DBSCAN'




        try:
            angle_max1, angle_max2 = \
            fs.fiber_detector_s4('DBSCAN', b1, b2, connectivity1,
connectivity2, mini1, mini2, \
                              eps1, eps2, tol_deg_in_cluster1,
tol_deg_out_cluster1, \
                              tol_deg_in_cluster2,
tol_deg_out_cluster2, \
                              w1_1, w2_1, w1_2, w2_2, image_augment1,
image_augment2, limit1, limit2)



            r1 = fs.viscosity_weight(angle_max1, last_angle1)
            r2 = fs.viscosity_weight(angle_max2, last_angle2)
            print('filter' + str(n) + ' worked')
            #print(angle_max1, r1, angle_max2, r2)
        except:
            print('filter' + str(n) + ' has compilling error')
            angle_max1 = last_angle1
            angle_max2 = last_angle2
            r1 = 0.00000001
```

```
            r2 = 0.00000001



        angle_weighted1 = angle_max1 * r1
        angle_weighted2 = angle_max2 * r2



        angle_weighted_sum1 = angle_weighted_sum1 + angle_weighted1
        w1 = w1 + r1
        angle_weighted_sum2 = angle_weighted_sum2 + angle_weighted2
        w2 = w2 + r2

    angle_weighted_ave1 = angle_weighted_sum1/(w1+0.0000000001)
    angle_weighted_ave2 = angle_weighted_sum2/(w2+0.0000000001)

    #final_angle1 = fs.viscosity_process(angle_weighted_ave1,
last_angle1)
    #final_angle2 = fs.viscosity_process(angle_weighted_ave2,
last_angle2)
    final_angle1 = angle_weighted_ave1
    final_angle2 = angle_weighted_ave2


    final_angle = final_angle1 + final_angle2
    print('final_angle: %2f'   %(final_angle))
    aug_visc.append(final_angle)



    last_angle1 = final_angle1
    last_angle2 = final_angle2
    last_angle = final_angle

    angle_weighted_sum1 = 0
    angle_weighted_sum2 = 0
    w1 = 0
    w2 = 0

    end_time = time.time()
    time_running = end_time - start_time
    time_visc.append(time_running)


    print('Running Time', time_running)
    print('')


    #print('aug_visc: %2f'   %(final_angle))
```

```
    print('')
    print('')

    #determine the starting & ending points


    print('----------------------')

print(case)
print(aug_visc)

#import csv

#csv_file =  open('angle_data.csv', 'w')
#csv_writer = csv.writer(csv_file)
#csv_writer.writerow(time_visc)
#csv_file.close()
```

```
from mycluster import Cluster_Process as cl
from mycluster import Fiber_Value as fiv
import math
import numpy as np
from numpy import *
from sklearn.cluster import KMeans
from sklearn.cluster import SpectralClustering
from sklearn.cluster import AgglomerativeClustering
from sklearn.cluster import DBSCAN

# cut off the edges
def denoising(image, threshold):
    image_new = np.zeros(image.shape)
    for x in range(0, image.shape[0]):
        for y in range(0, image.shape[1]):
            if image[x, y] >= threshold:
                image_new[x, y] = image[x, y]
    return image_new


def cut_image(image, cut_position, cut_modifier, right_max, down_max):

    #trim the images
    image1 = image[1:cut_modifier+cut_position, 1:right_max]
    image2 = image[cut_modifier+cut_position:down_max, 1:right_max]

    #convert image type
    image1 = image1.astype(np.uint8)
    image2 = image2.astype(np.uint8)

    return image1, image2



def trim_image(image, left, right, up, down):

    #trim the images
    image = image[down:up, left:right]

    return image



def find_cut_position(image_zoom_clean):
    row = image_zoom_clean.shape[0]
    col = image_zoom_clean.shape[1]

    temp_array = []

    for i in range(1, row):
        for j in range (1, col):
            if image_zoom_clean[i][j] > 0:
                temp_array.append(i)
                break
    cut_position = int((min(temp_array) + max(temp_array))/2)
    return cut_position


def find_cut_position_pkg(image, filter_percent, left_inner, right_inner, up_inner, down_inner):
    #image_t = trim_image(image, left_most, right_most, down_most, up_most)
    image_zoom= image.astype(np.uint8)
    #image_zoom = image_t_t[down_most:up_most, left_most:right_most]
    threshold = np.percentile(image_zoom, filter_percent)
    image_zoom_clean = cl.denoising(image_zoom, threshold)
    image_zoom_clean = np.array(image_zoom_clean, dtype=np.uint8)
    cut_position = find_cut_position(image_zoom_clean) + 1

    return cut_position


def find_cut_position_pkg2(image, filter_intensity, left_inner, right_inner, up_inner, down_inner):
    #image_t = trim_image(image, left_most, right_most, down_most, up_most)
    image_zoom= image.astype(np.uint8)
    #image_zoom = image_t_t[down_most:up_most, left_most:right_most]
    image_zoom_clean = cl.denoising(image_zoom, filter_intensity)
    image_zoom_clean = np.array(image_zoom_clean, dtype=np.uint8)
    cut_position = find_cut_position(image_zoom_clean) + 1
```

```
    return cut_position



def find_cut_position_pkg_simple(image, filter_percent):
    image_t1 = cut_image_o(image)
    image_t2 = image_t1.astype(np.uint8)
    image_zoom = image_t2[140:180, 400:500]
    threshold = np.percentile(image_zoom, filter_percent)
    image_zoom_clean = cl.denoising(image_zoom, threshold)
    image_zoom_clean = np.array(image_zoom_clean, dtype=np.uint8)
    cut_position = find_cut_position(image_zoom_clean)

    return cut_position


def cut_image_general(image, trim_ver, trim_ver_start, trim_hor, basic_cut, cut_position):

    #trim the images
    image1 = image[trim_ver_start:basic_cut + cut_position, 1:trim_hor]
    image2 = image[basic_cut + cut_position + 1:trim_ver, 1:trim_hor]

    #convert image type
    image1 = image1.astype(np.uint8)
    image2 = image2.astype(np.uint8)

    return image1, image2

# detect the muscle fiber that has the highest value





# detect the muscle fiber that has the highest value-----only get the average angle_mix cw and no-cw
def fiber_detector(method, b1, b2, connectivity1, connectivity2, n_clusters1, n_clusters2, \
                   eps1, eps2, tol_deg_in_cluster1, tol_deg_out_cluster1, \
                   tol_deg_in_cluster2, tol_deg_out_cluster2, \
                   w1_1, w2_1, w1_2, w2_2, image1, image2):



    ## Import the images
    #denoising
    threshold1 = np.percentile(image1,b1)
    image_new1 = cl.denoising(image1, threshold1)

    threshold2 = np.percentile(image2,b2)
    image_new2 = cl.denoising(image2, threshold2)

    #convert image type
    image_new1 = np.array(image_new1, dtype=np.uint8)
    image_new2 = np.array(image_new2, dtype=np.uint8)

    #get the clean images
    #image_new_clean1 = cl.get_clean_image(image_new1, connectivity1)
    #image_new_clean2 = cl.get_clean_image(image_new2, connectivity2)
    image_new_clean1 = image_new1
    image_new_clean2 = image_new2

    ## Cluster the imges and detect the fibers
    #get the pixel locations
    threshold_standoutcluster = 0.1
    pixel_position1 = cl.pixel_location(image_new_clean1, threshold_standoutcluster)
    pixel_position2 = cl.pixel_location(image_new_clean2, threshold_standoutcluster)

    #apply cluster method



    if method == 'KMeans':

        k_instance_kmean1 = \
        KMeans(max_iter=100, n_clusters = n_clusters1, init='k-means++').fit(pixel_position1)
        k_instance_kmean2 = \
        KMeans(max_iter=100, n_clusters = n_clusters2, init='k-means++').fit(pixel_position2)
```

```
        cluster_label1 = k_instance_kmean1.labels_
        cluster_label2 = k_instance_kmean2.labels_
        cluster_num1 = k_instance_kmean1.cluster_centers_.shape[0]
        cluster_num2 = k_instance_kmean2.cluster_centers_.shape[0]


    if method == 'SpectralClustering':

        spectral_model_rbf1 = SpectralClustering(n_clusters = n_clusters1, affinity ='rbf')
        spectral_model_rbf2 = SpectralClustering(n_clusters = n_clusters2, affinity ='rbf')

        labels_rbf1 = \
        spectral_model_rbf1.fit_predict(pixel_position1)
        labels_rbf2 = \
        spectral_model_rbf2.fit_predict(pixel_position2)

        cluster_label1 = labels_rbf1
        cluster_label2 = labels_rbf2
        cluster_num1 = labels_rbf1.max()
        cluster_num2 = labels_rbf2.max()


    if method == "AgglomerativeClustering":

        cluster_agg1 = AgglomerativeClustering(n_clusters = n_clusters1).fit(pixel_position1)
        cluster_agg2 = AgglomerativeClustering(n_clusters = n_clusters2).fit(pixel_position2)

        cluster_label1 = cluster_agg1.labels_
        cluster_label2 = cluster_agg2.labels_
        cluster_num1 = cluster_label1.max()
        cluster_num2 = cluster_label2.max()


    if method == "DBSCAN":

        cluster_DBSCAN1 = DBSCAN(eps = eps1, min_samples=n_clusters1).fit(pixel_position1)
        cluster_DBSCAN2 = DBSCAN(eps = eps2, min_samples=n_clusters2).fit(pixel_position2)

        cluster_label1 = cluster_DBSCAN1.labels_
        cluster_label2 = cluster_DBSCAN2.labels_

        cluster_num1 = cluster_label1.max()
        cluster_num2 = cluster_label2.max()


    #cluster
    cluster_dict1 = cl.pixel_cluster(cluster_label1, pixel_position1, cluster_num1)
    cluster_dict2 = cl.pixel_cluster(cluster_label2, pixel_position2, cluster_num2)

    #find the vertices
    right_down_dict1, right_up_dict1, left_down_dict1, left_up_dict1 \
    = cl.cluster_vertex(cluster_num1, cluster_dict1)
    right_down_dict2, right_up_dict2, left_down_dict2, left_up_dict2 \
    = cl.cluster_vertex(cluster_num2, cluster_dict2)

    #find clusters angles
    cluster_angles1 = cl.cluster_angle_fun(cluster_num1, cluster_dict1, \
                                    right_down_dict1, right_up_dict1, \
                                    left_down_dict1, left_up_dict1)
    cluster_angles2 = cl.cluster_angle_fun(cluster_num2, cluster_dict2, \
                                    right_down_dict2, right_up_dict2, \
                                    left_down_dict2, left_up_dict2)

    #find fibers angles
    tol_in_cluster1 = tol_deg_in_cluster1*pi/180
    tol_out_cluster1 = tol_deg_in_cluster1*pi/180
    tol_in_cluster2 = tol_deg_in_cluster2*pi/180
    tol_out_cluster2 = tol_deg_in_cluster2*pi/180


    cluster_dict_new1, right_down_dict_new1, right_up_dict_new1, \
    left_down_dict_new1, left_up_dict_new1 \
    = cl.cluster_update(cluster_num1, cluster_angles1, tol_in_cluster1, tol_out_cluster1, \
                    cluster_dict1, right_down_dict1, right_up_dict1, \
                    left_down_dict1, left_up_dict1)
```

```
    cluster_dict_new2, right_down_dict_new2, right_up_dict_new2, \
    left_down_dict_new2, left_up_dict_new2 \
    = cl.cluster_update(cluster_num2, cluster_angles2, tol_in_cluster2, tol_out_cluster2, \
                     cluster_dict2, right_down_dict2, right_up_dict2, \
                     left_down_dict2, left_up_dict2)

    cluster_angle_new1 = cl.cluster_angle_fun(cluster_num1, cluster_dict_new1, \
                                       right_down_dict_new1, right_up_dict_new1, \
                                       left_down_dict_new1, left_up_dict_new1)

    cluster_angle_new2 = cl.cluster_angle_fun(cluster_num2, cluster_dict_new2, \
                                       right_down_dict_new2, right_up_dict_new2, \
                                       left_down_dict_new2, left_up_dict_new2)

    cluster_dict_new_posi1, cluster_dict_new_nega1, right_down_dict_posi1, right_up_dict_posi1, \
    left_down_dict_posi1, left_up_dict_posi1, right_down_dict_nega1, right_up_dict_nega1, \
    left_down_dict_nega1, left_up_dict_nega1\
    = cl.cluster_group(cluster_num1, cluster_dict_new1, cluster_angle_new1, \
        right_down_dict_new1, right_up_dict_new1, \
                left_down_dict_new1, left_up_dict_new1)

    cluster_dict_new_posi2, cluster_dict_new_nega2, right_down_dict_posi2, right_up_dict_posi2, \
    left_down_dict_posi2, left_up_dict_posi2, right_down_dict_nega2, right_up_dict_nega2, \
    left_down_dict_nega2, left_up_dict_nega2 \
    = cl.cluster_group(cluster_num2, cluster_dict_new2, cluster_angle_new2, \
        right_down_dict_new2, right_up_dict_new2, \
                left_down_dict_new2, left_up_dict_new2)




    ## Selelct the muscle fibers

    #define the angle sets that will be used for upper pic
    cluster_dict_new1, right_down_dict1, right_up_dict1, left_down_dict1, left_up_dict1 = \
    cluster_dict_new_nega1, right_down_dict_nega1, right_up_dict_nega1, \
    left_down_dict_nega1, left_up_dict_nega1

    #define the angle sets that will be used for downner pic
    cluster_dict_new2, right_down_dict2, right_up_dict2, left_down_dict2, left_up_dict2 = \
    cluster_dict_new_posi2, right_down_dict_posi2, right_up_dict_posi2, \
    left_down_dict_posi2, left_up_dict_posi2



    value_dict1 = fiv.Value_Funtion(1, w1_1, w2_1, image_new_clean1, \
                              cluster_dict_new1, right_down_dict1, right_up_dict1, \
                              left_down_dict1, left_up_dict1)

    index1, value1 = fiv.select_high_value(value_dict1)


    right_pixel1, left_pixel1 = fiv.find_vertices(index1, right_down_dict1, left_up_dict1)


    value_dict2 = fiv.Value_Funtion(2, w1_2, w2_2, image_new_clean2, \
                              cluster_dict_new2, right_down_dict2, right_up_dict2, \
                              left_down_dict2, left_up_dict2)

    index2, value2 = fiv.select_high_value(value_dict2)

    right_pixel2, left_pixel2 = fiv.find_vertices(index2, right_up_dict2, left_down_dict2)


    hor_l, hor_r = np.array([45, 1]), np.array([45, 5])

    #angle selected based on the max value
    angle_max1 = angle_compute(right_pixel1, left_pixel1, hor_r, hor_l)
    angle_max2 = angle_compute(right_pixel2, left_pixel2, hor_r, hor_l)

    angle_max = angle_max1 + angle_max2

    return angle_max
```

```
# detect the muscle fiber that has the highest value-----only get the average angle_mix cw and no-cw
def fiber_detector_s(method, b1, b2, connectivity1, connectivity2, n_clusters1, n_clusters2, \
                   eps1, eps2, tol_deg_in_cluster1, tol_deg_out_cluster1, \
                   tol_deg_in_cluster2, tol_deg_out_cluster2, \
                   w1_1, w2_1, w1_2, w2_2, image1, image2):



    ## Import the images
    #denoising
    threshold1 = np.percentile(image1,b1)
    image_new1 = cl.denoising(image1, threshold1)

    threshold2 = np.percentile(image2,b2)
    image_new2 = cl.denoising(image2, threshold2)

    #convert image type
    image_new1 = np.array(image_new1, dtype=np.uint8)
    image_new2 = np.array(image_new2, dtype=np.uint8)

    #get the clean images
    #image_new_clean1 = cl.get_clean_image(image_new1, connectivity1)
    #image_new_clean2 = cl.get_clean_image(image_new2, connectivity2)
    image_new_clean1 = image_new1
    image_new_clean2 = image_new2

    ## Cluster the imges and detect the fibers
    #get the pixel locations
    threshold_standoutcluster = 0.1
    pixel_position1 = cl.pixel_location(image_new_clean1, threshold_standoutcluster)
    pixel_position2 = cl.pixel_location(image_new_clean2, threshold_standoutcluster)

    #apply cluster method
    #apply k-means



    if method == 'KMeans':

        k_instance_kmean1 = \
        KMeans(max_iter=100, n_clusters = n_clusters1, init='k-means++').fit(pixel_position1)
        k_instance_kmean2 = \
        KMeans(max_iter=100, n_clusters = n_clusters2, init='k-means++').fit(pixel_position2)

        cluster_label1 = k_instance_kmean1.labels_
        cluster_label2 = k_instance_kmean2.labels_
        cluster_num1 = k_instance_kmean1.cluster_centers_.shape[0]
        cluster_num2 = k_instance_kmean2.cluster_centers_.shape[0]


    if method == 'SpectralClustering':

        spectral_model_rbf1 = SpectralClustering(n_clusters = n_clusters1, affinity ='rbf')
        spectral_model_rbf2 = SpectralClustering(n_clusters = n_clusters2, affinity ='rbf')

        labels_rbf1 = \
        spectral_model_rbf1.fit_predict(pixel_position1)
        labels_rbf2 = \
        spectral_model_rbf2.fit_predict(pixel_position2)

        cluster_label1 = labels_rbf1
        cluster_label2 = labels_rbf2
        cluster_num1 = labels_rbf1.max()
        cluster_num2 = labels_rbf2.max()


    if method == "AgglomerativeClustering":

        cluster_agg1 = AgglomerativeClustering(n_clusters = n_clusters1).fit(pixel_position1)
        cluster_agg2 = AgglomerativeClustering(n_clusters = n_clusters2).fit(pixel_position2)
```

```
        cluster_label1 = cluster_agg1.labels_
        cluster_label2 = cluster_agg2.labels_
        cluster_num1 = cluster_label1.max()
        cluster_num2 = cluster_label2.max()


    if method == "DBSCAN":

        cluster_DBSCAN1 = DBSCAN(eps = eps1, min_samples=n_clusters1).fit(pixel_position1)
        cluster_DBSCAN2 = DBSCAN(eps = eps2, min_samples=n_clusters2).fit(pixel_position2)

        cluster_label1 = cluster_DBSCAN1.labels_
        cluster_label2 = cluster_DBSCAN2.labels_

        cluster_num1 = cluster_label1.max()
        cluster_num2 = cluster_label2.max()


    #cluster
    cluster_dict1 = cl.pixel_cluster(cluster_label1, pixel_position1, cluster_num1)
    cluster_dict2 = cl.pixel_cluster(cluster_label2, pixel_position2, cluster_num2)

    #find the vertices
    right_down_dict1, right_up_dict1, left_down_dict1, left_up_dict1 \
    = cl.cluster_vertex(cluster_num1, cluster_dict1)
    right_down_dict2, right_up_dict2, left_down_dict2, left_up_dict2 \
    = cl.cluster_vertex(cluster_num2, cluster_dict2)

    #find clusters angles
    cluster_angles1 = cl.cluster_angle_fun(cluster_num1, cluster_dict1, \
                                    right_down_dict1, right_up_dict1, \
                                    left_down_dict1, left_up_dict1)
    cluster_angles2 = cl.cluster_angle_fun(cluster_num2, cluster_dict2, \
                                    right_down_dict2, right_up_dict2, \
                                    left_down_dict2, left_up_dict2)

    #find fibers angles
    tol_in_cluster1 = tol_deg_in_cluster1*pi/180
    tol_out_cluster1 = tol_deg_in_cluster1*pi/180
    tol_in_cluster2 = tol_deg_in_cluster2*pi/180
    tol_out_cluster2 = tol_deg_in_cluster2*pi/180


    cluster_dict_new1, right_down_dict_new1, right_up_dict_new1, \
    left_down_dict_new1, left_up_dict_new1 \
    = cl.cluster_update(cluster_num1, cluster_angles1, tol_in_cluster1, tol_out_cluster1, \
                    cluster_dict1, right_down_dict1, right_up_dict1, \
                    left_down_dict1, left_up_dict1)

    cluster_dict_new2, right_down_dict_new2, right_up_dict_new2, \
    left_down_dict_new2, left_up_dict_new2 \
    = cl.cluster_update(cluster_num2, cluster_angles2, tol_in_cluster2, tol_out_cluster2, \
                    cluster_dict2, right_down_dict2, right_up_dict2, \
                    left_down_dict2, left_up_dict2)

    cluster_angle_new1 = cl.cluster_angle_fun(cluster_num1, cluster_dict_new1, \
                                       right_down_dict_new1, right_up_dict_new1, \
                                       left_down_dict_new1, left_up_dict_new1)

    cluster_angle_new2 = cl.cluster_angle_fun(cluster_num2, cluster_dict_new2, \
                                       right_down_dict_new2, right_up_dict_new2, \
                                       left_down_dict_new2, left_up_dict_new2)

    cluster_dict_new_posi1, cluster_dict_new_nega1, right_down_dict_posi1, right_up_dict_posi1, \
    left_down_dict_posi1, left_up_dict_posi1, right_down_dict_nega1, right_up_dict_nega1, \
    left_down_dict_nega1, left_up_dict_nega1\
    = cl.cluster_group(cluster_num1, cluster_dict_new1, cluster_angle_new1, \
        right_down_dict_new1, right_up_dict_new1, \
                    left_down_dict_new1, left_up_dict_new1)

    cluster_dict_new_posi2, cluster_dict_new_nega2, right_down_dict_posi2, right_up_dict_posi2, \
    left_down_dict_posi2, left_up_dict_posi2, right_down_dict_nega2, right_up_dict_nega2, \
    left_down_dict_nega2, left_up_dict_nega2 \
    = cl.cluster_group(cluster_num2, cluster_dict_new2, cluster_angle_new2, \
        right_down_dict_new2, right_up_dict_new2, \
                    left_down_dict_new2, left_up_dict_new2)
```

```
    ## Selelct the muscle fibers

    #define the angle sets that will be used for upper pic
    cluster_dict_new1, right_down_dict1, right_up_dict1, left_down_dict1, left_up_dict1 = \
    cluster_dict_new_nega1, right_down_dict_nega1, right_up_dict_nega1, \
    left_down_dict_nega1, left_up_dict_nega1

    #define the angle sets that will be used for downner pic
    cluster_dict_new2, right_down_dict2, right_up_dict2, left_down_dict2, left_up_dict2 = \
    cluster_dict_new_posi2, right_down_dict_posi2, right_up_dict_posi2, \
    left_down_dict_posi2, left_up_dict_posi2



    value_dict1 = fiv.Value_Funtion(1, w1_1, w2_1, image_new_clean1, \
                              cluster_dict_new1, right_down_dict1, right_up_dict1, \
                              left_down_dict1, left_up_dict1)

    index1, value1 = fiv.select_high_value(value_dict1)


    right_pixel1, left_pixel1 = fiv.find_vertices(index1, right_down_dict1, left_up_dict1)


    value_dict2 = fiv.Value_Funtion(2, w1_2, w2_2, image_new_clean2, \
                              cluster_dict_new2, right_down_dict2, right_up_dict2, \
                              left_down_dict2, left_up_dict2)

    index2, value2 = fiv.select_high_value(value_dict2)

    right_pixel2, left_pixel2 = fiv.find_vertices(index2, right_up_dict2, left_down_dict2)


    hor_l, hor_r = np.array([45, 1]), np.array([45, 5])

    #angle selected based on the max value
    angle_max1 = angle_compute(right_pixel1, left_pixel1, hor_r, hor_l)
    angle_max2 = angle_compute(right_pixel2, left_pixel2, hor_r, hor_l)




    return angle_max1, angle_max2



def fiber_detector_s2(method, b1, b2, connectivity1, connectivity2, n_clusters1, n_clusters2, \
               eps1, eps2, tol_deg_in_cluster1, tol_deg_out_cluster1, \
               tol_deg_in_cluster2, tol_deg_out_cluster2, \
               w1_1, w2_1, w1_2, w2_2, image1, image2, last_angle1, last_angle2, sigma1, sigma2, limit1, limit2



    ## Import the images
    #denoising
    threshold1 = np.percentile(image1,b1)
    image_new1 = cl.denoising(image1, threshold1)

    threshold2 = np.percentile(image2,b2)
    image_new2 = cl.denoising(image2, threshold2)

    #convert image type
    image_new1 = np.array(image_new1, dtype=np.uint8)
    image_new2 = np.array(image_new2, dtype=np.uint8)

    #get the clean images
    #image_new_clean1 = cl.get_clean_image(image_new1, connectivity1)
    #image_new_clean2 = cl.get_clean_image(image_new2, connectivity2)
    image_new_clean1 = image_new1
    image_new_clean2 = image_new2

    ## Cluster the imges and detect the fibers
    #get the pixel locations
```

```
threshold_standoutcluster = 0.1
pixel_position1 = cl.pixel_location(image_new_clean1, threshold_standoutcluster)
pixel_position2 = cl.pixel_location(image_new_clean2, threshold_standoutcluster)

#apply cluster method
#apply k-means



if method == 'KMeans':

    k_instance_kmean1 = \
    KMeans(max_iter=100, n_clusters = n_clusters1, init='k-means++').fit(pixel_position1)
    k_instance_kmean2 = \
    KMeans(max_iter=100, n_clusters = n_clusters2, init='k-means++').fit(pixel_position2)

    cluster_label1 = k_instance_kmean1.labels_
    cluster_label2 = k_instance_kmean2.labels_
    cluster_num1 = k_instance_kmean1.cluster_centers_.shape[0]
    cluster_num2 = k_instance_kmean2.cluster_centers_.shape[0]


if method == 'SpectralClustering':

    spectral_model_rbf1 = SpectralClustering(n_clusters = n_clusters1, affinity ='rbf')
    spectral_model_rbf2 = SpectralClustering(n_clusters = n_clusters2, affinity ='rbf')

    labels_rbf1 = \
    spectral_model_rbf1.fit_predict(pixel_position1)
    labels_rbf2 = \
    spectral_model_rbf2.fit_predict(pixel_position2)

    cluster_label1 = labels_rbf1
    cluster_label2 = labels_rbf2
    cluster_num1 = labels_rbf1.max()
    cluster_num2 = labels_rbf2.max()


if method == "AgglomerativeClustering":

    cluster_agg1 = AgglomerativeClustering(n_clusters = n_clusters1).fit(pixel_position1)
    cluster_agg2 = AgglomerativeClustering(n_clusters = n_clusters2).fit(pixel_position2)

    cluster_label1 = cluster_agg1.labels_
    cluster_label2 = cluster_agg2.labels_
    cluster_num1 = cluster_label1.max()
    cluster_num2 = cluster_label2.max()


if method == "DBSCAN":

    cluster_DBSCAN1 = DBSCAN(eps = eps1, min_samples=n_clusters1).fit(pixel_position1)
    cluster_DBSCAN2 = DBSCAN(eps = eps2, min_samples=n_clusters2).fit(pixel_position2)

    cluster_label1 = cluster_DBSCAN1.labels_
    cluster_label2 = cluster_DBSCAN2.labels_

    cluster_num1 = cluster_label1.max()
    cluster_num2 = cluster_label2.max()


#cluster
cluster_dict1 = cl.pixel_cluster(cluster_label1, pixel_position1, cluster_num1)
cluster_dict2 = cl.pixel_cluster(cluster_label2, pixel_position2, cluster_num2)

#find the vertices
right_down_dict1, right_up_dict1, left_down_dict1, left_up_dict1 \
= cl.cluster_vertex(cluster_num1, cluster_dict1)
right_down_dict2, right_up_dict2, left_down_dict2, left_up_dict2 \
= cl.cluster_vertex(cluster_num2, cluster_dict2)

#find clusters angles
cluster_angles1 = cl.cluster_angle_fun(cluster_num1, cluster_dict1, \
                                    right_down_dict1, right_up_dict1, \
                                    left_down_dict1, left_up_dict1)
cluster_angles2 = cl.cluster_angle_fun(cluster_num2, cluster_dict2, \
                                    right_down_dict2, right_up_dict2, \
```

```
                                                    left_down_dict2, left_up_dict2)

    #find fibers angles
    tol_in_cluster1 = tol_deg_in_cluster1*pi/180
    tol_out_cluster1 = tol_deg_in_cluster1*pi/180
    tol_in_cluster2 = tol_deg_in_cluster2*pi/180
    tol_out_cluster2 = tol_deg_in_cluster2*pi/180


    cluster_dict_new1, right_down_dict_new1, right_up_dict_new1, \
    left_down_dict_new1, left_up_dict_new1 \
    = cl.cluster_update(cluster_num1, cluster_angles1, tol_in_cluster1, tol_out_cluster1, \
                     cluster_dict1, right_down_dict1, right_up_dict1, \
                     left_down_dict1, left_up_dict1)

    cluster_dict_new2, right_down_dict_new2, right_up_dict_new2, \
    left_down_dict_new2, left_up_dict_new2 \
    = cl.cluster_update(cluster_num2, cluster_angles2, tol_in_cluster2, tol_out_cluster2, \
                     cluster_dict2, right_down_dict2, right_up_dict2, \
                     left_down_dict2, left_up_dict2)

    cluster_angle_new1 = cl.cluster_angle_fun(cluster_num1, cluster_dict_new1, \
                                          right_down_dict_new1, right_up_dict_new1, \
                                          left_down_dict_new1, left_up_dict_new1)

    cluster_angle_new2 = cl.cluster_angle_fun(cluster_num2, cluster_dict_new2, \
                                          right_down_dict_new2, right_up_dict_new2, \
                                          left_down_dict_new2, left_up_dict_new2)

    cluster_dict_new_posi1, cluster_dict_new_nega1, right_down_dict_posi1, right_up_dict_posi1, \
    left_down_dict_posi1, left_up_dict_posi1, right_down_dict_nega1, right_up_dict_nega1, \
    left_down_dict_nega1, left_up_dict_nega1\
    = cl.cluster_group(cluster_num1, cluster_dict_new1, cluster_angle_new1, \
        right_down_dict_new1, right_up_dict_new1, \
                    left_down_dict_new1, left_up_dict_new1)

    cluster_dict_new_posi2, cluster_dict_new_nega2, right_down_dict_posi2, right_up_dict_posi2, \
    left_down_dict_posi2, left_up_dict_posi2, right_down_dict_nega2, right_up_dict_nega2, \
    left_down_dict_nega2, left_up_dict_nega2 \
    = cl.cluster_group(cluster_num2, cluster_dict_new2, cluster_angle_new2, \
        right_down_dict_new2, right_up_dict_new2, \
                    left_down_dict_new2, left_up_dict_new2)




    ## Selelct the muscle fibers

    #define the angle sets that will be used for upper pic
    cluster_dict_new1, right_down_dict1, right_up_dict1, left_down_dict1, left_up_dict1 = \
    cluster_dict_new_nega1, right_down_dict_nega1, right_up_dict_nega1, \
    left_down_dict_nega1, left_up_dict_nega1

    #define the angle sets that will be used for downner pic
    cluster_dict_new2, right_down_dict2, right_up_dict2, left_down_dict2, left_up_dict2 = \
    cluster_dict_new_posi2, right_down_dict_posi2, right_up_dict_posi2, \
    left_down_dict_posi2, left_up_dict_posi2



    value_dict1 = fiv.Value_Funtion(1, w1_1, w2_1, image_new_clean1, \
                              cluster_dict_new1, right_down_dict1, right_up_dict1, \
                              left_down_dict1, left_up_dict1)

    index1, value1 = fiv.select_high_value(value_dict1)


    right_pixel1, left_pixel1 = fiv.find_vertices(index1, right_down_dict1, left_up_dict1)


    value_dict2 = fiv.Value_Funtion(2, w1_2, w2_2, image_new_clean2, \
                              cluster_dict_new2, right_down_dict2, right_up_dict2, \
                              left_down_dict2, left_up_dict2)

    index2, value2 = fiv.select_high_value(value_dict2)

    right_pixel2, left_pixel2 = fiv.find_vertices(index2, right_up_dict2, left_down_dict2)
```

```
    hor_l, hor_r = np.array([45, 1]), np.array([45, 5])

    #angle selected based on the max value
    angle_max1 = angle_compute(right_pixel1, left_pixel1, hor_r, hor_l)
    angle_max2 = angle_compute(right_pixel2, left_pixel2, hor_r, hor_l)



    #angle selected based on the average value
    angle_ave1 = angle_from_values(value_dict1, right_down_dict1, left_up_dict1)
    angle_ave2 = angle_from_values(value_dict2, right_down_dict2, left_up_dict2)




    #angle selected based on the weighted average value
    angle_ave_cw1 = angle_from_values_cw(value_dict1, right_down_dict1, left_up_dict1, angle_max1, sigma1)
    angle_ave_cw2 = angle_from_values_cw(value_dict2, right_down_dict2, left_up_dict2, angle_max2, sigma2)




    return angle_max1, angle_max2, angle_ave1, angle_ave2, angle_ave_cw1, angle_ave_cw2


def fiber_detector_s3(method, b1, b2, connectivity1, connectivity2, n_clusters1, n_clusters2, \
               eps1, eps2, tol_deg_in_cluster1, tol_deg_out_cluster1, \
               tol_deg_in_cluster2, tol_deg_out_cluster2, \
               w1_1, w2_1, w1_2, w2_2, image1, image2, limit1, limit2):



    ## Import the images
    #denoising
    #threshold1 = np.percentile(image1,b1)
    image_new1 = cl.denoising2(image1, b1)

    #threshold2 = np.percentile(image2,b2)
    image_new2 = cl.denoising2(image2, b2)

    #convert image type
    image_new1 = np.array(image_new1, dtype=np.uint8)
    image_new2 = np.array(image_new2, dtype=np.uint8)

    #get the clean images
    #image_new_clean1 = cl.get_clean_image(image_new1, connectivity1)
    #image_new_clean2 = cl.get_clean_image(image_new2, connectivity2)
    image_new_clean1 = image_new1
    image_new_clean2 = image_new2

    ## Cluster the imges and detect the fibers
    #get the pixel locations
    threshold_standoutcluster = 0.1
    pixel_position1 = cl.pixel_location(image_new_clean1, threshold_standoutcluster)
    pixel_position2 = cl.pixel_location(image_new_clean2, threshold_standoutcluster)

    #apply cluster method
    #apply k-means



    if method == 'KMeans':

        k_instance_kmean1 = \
        KMeans(max_iter=100, n_clusters = n_clusters1, init='k-means++').fit(pixel_position1)
        k_instance_kmean2 = \
        KMeans(max_iter=100, n_clusters = n_clusters2, init='k-means++').fit(pixel_position2)

        cluster_label1 = k_instance_kmean1.labels_
        cluster_label2 = k_instance_kmean2.labels_
        cluster_num1 = k_instance_kmean1.cluster_centers_.shape[0]
        cluster_num2 = k_instance_kmean2.cluster_centers_.shape[0]


    if method == 'SpectralClustering':
```

```
        spectral_model_rbf1 = SpectralClustering(n_clusters = n_clusters1, affinity ='rbf')
        spectral_model_rbf2 = SpectralClustering(n_clusters = n_clusters2, affinity ='rbf')

        labels_rbf1 = \
        spectral_model_rbf1.fit_predict(pixel_position1)
        labels_rbf2 = \
        spectral_model_rbf2.fit_predict(pixel_position2)

        cluster_label1 = labels_rbf1
        cluster_label2 = labels_rbf2
        cluster_num1 = labels_rbf1.max()
        cluster_num2 = labels_rbf2.max()


    if method == "AgglomerativeClustering":

        cluster_agg1 = AgglomerativeClustering(n_clusters = n_clusters1).fit(pixel_position1)
        cluster_agg2 = AgglomerativeClustering(n_clusters = n_clusters2).fit(pixel_position2)

        cluster_label1 = cluster_agg1.labels_
        cluster_label2 = cluster_agg2.labels_
        cluster_num1 = cluster_label1.max()
        cluster_num2 = cluster_label2.max()


    if method == "DBSCAN":

        cluster_DBSCAN1 = DBSCAN(eps = eps1, min_samples=n_clusters1).fit(pixel_position1)
        cluster_DBSCAN2 = DBSCAN(eps = eps2, min_samples=n_clusters2).fit(pixel_position2)

        cluster_label1 = cluster_DBSCAN1.labels_
        cluster_label2 = cluster_DBSCAN2.labels_

        cluster_num1 = cluster_label1.max()
        cluster_num2 = cluster_label2.max()


    #cluster
    cluster_dict1, cluster_num_new1 = cl.pixel_cluster2(cluster_label1, pixel_position1, cluster_num1, limit1)
    cluster_dict2, cluster_num_new2 = cl.pixel_cluster2(cluster_label2, pixel_position2, cluster_num2, limit2)

    cluster_num1 = cluster_num_new1
    cluster_num2 = cluster_num_new2

    #find the vertices
    right_down_dict1, right_up_dict1, left_down_dict1, left_up_dict1 \
    = cl.cluster_vertex(cluster_num1, cluster_dict1)
    right_down_dict2, right_up_dict2, left_down_dict2, left_up_dict2 \
    = cl.cluster_vertex(cluster_num2, cluster_dict2)

    #find clusters angles
    cluster_angles1 = cl.cluster_angle_fun(cluster_num1, cluster_dict1, \
                                    right_down_dict1, right_up_dict1, \
                                    left_down_dict1, left_up_dict1)
    cluster_angles2 = cl.cluster_angle_fun(cluster_num2, cluster_dict2, \
                                    right_down_dict2, right_up_dict2, \
                                    left_down_dict2, left_up_dict2)

    #find fibers angles
    tol_in_cluster1 = tol_deg_in_cluster1*pi/180
    tol_out_cluster1 = tol_deg_in_cluster1*pi/180
    tol_in_cluster2 = tol_deg_in_cluster2*pi/180
    tol_out_cluster2 = tol_deg_in_cluster2*pi/180


    cluster_dict_new1, right_down_dict_new1, right_up_dict_new1, \
    left_down_dict_new1, left_up_dict_new1 \
    = cl.cluster_update(cluster_num1, cluster_angles1, tol_in_cluster1, tol_out_cluster1, \
                  cluster_dict1, right_down_dict1, right_up_dict1, \
                  left_down_dict1, left_up_dict1)

    cluster_dict_new2, right_down_dict_new2, right_up_dict_new2, \
    left_down_dict_new2, left_up_dict_new2 \
    = cl.cluster_update(cluster_num2, cluster_angles2, tol_in_cluster2, tol_out_cluster2, \
                  cluster_dict2, right_down_dict2, right_up_dict2, \
                  left_down_dict2, left_up_dict2)
```

```
    cluster_angle_new1 = cl.cluster_angle_fun(cluster_num1, cluster_dict_new1, \
                                       right_down_dict_new1, right_up_dict_new1, \
                                       left_down_dict_new1, left_up_dict_new1)

    cluster_angle_new2 = cl.cluster_angle_fun(cluster_num2, cluster_dict_new2, \
                                       right_down_dict_new2, right_up_dict_new2, \
                                       left_down_dict_new2, left_up_dict_new2)

    cluster_dict_new_posi1, cluster_dict_new_nega1, right_down_dict_posi1, right_up_dict_posi1, \
    left_down_dict_posi1, left_up_dict_posi1, right_down_dict_nega1, right_up_dict_nega1, \
    left_down_dict_nega1, left_up_dict_nega1\
    = cl.cluster_group(cluster_num1, cluster_dict_new1, cluster_angle_new1, \
        right_down_dict_new1, right_up_dict_new1, \
                    left_down_dict_new1, left_up_dict_new1)

    cluster_dict_new_posi2, cluster_dict_new_nega2, right_down_dict_posi2, right_up_dict_posi2, \
    left_down_dict_posi2, left_up_dict_posi2, right_down_dict_nega2, right_up_dict_nega2, \
    left_down_dict_nega2, left_up_dict_nega2 \
    = cl.cluster_group(cluster_num2, cluster_dict_new2, cluster_angle_new2, \
        right_down_dict_new2, right_up_dict_new2, \
                    left_down_dict_new2, left_up_dict_new2)




    ## Selelct the muscle fibers

    #define the angle sets that will be used for upper pic
    cluster_dict_new1, right_down_dict1, right_up_dict1, left_down_dict1, left_up_dict1 = \
    cluster_dict_new_nega1, right_down_dict_nega1, right_up_dict_nega1, \
    left_down_dict_nega1, left_up_dict_nega1

    #define the angle sets that will be used for downner pic
    cluster_dict_new2, right_down_dict2, right_up_dict2, left_down_dict2, left_up_dict2 = \
    cluster_dict_new_posi2, right_down_dict_posi2, right_up_dict_posi2, \
    left_down_dict_posi2, left_up_dict_posi2



    value_dict1 = fiv.Value_Funtion(1, w1_1, w2_1, image_new_clean1, \
                              cluster_dict_new1, right_down_dict1, right_up_dict1, \
                              left_down_dict1, left_up_dict1)

    index1, value1 = fiv.select_high_value(value_dict1)


    right_pixel1, left_pixel1 = fiv.find_vertices(index1, right_down_dict1, left_up_dict1)


    value_dict2 = fiv.Value_Funtion(2, w1_2, w2_2, image_new_clean2, \
                              cluster_dict_new2, right_down_dict2, right_up_dict2, \
                              left_down_dict2, left_up_dict2)

    index2, value2 = fiv.select_high_value(value_dict2)

    right_pixel2, left_pixel2 = fiv.find_vertices(index2, right_up_dict2, left_down_dict2)


    hor_l, hor_r = np.array([45, 1]), np.array([45, 5])

    #angle selected based on the max value
    angle_max1 = angle_compute(right_pixel1, left_pixel1, hor_r, hor_l)
    angle_max2 = angle_compute(right_pixel2, left_pixel2, hor_r, hor_l)





    return angle_max1, angle_max2



def fiber_detector_s4(method, b1, b2, connectivity1, connectivity2, n_clusters1, n_clusters2, \
                  eps1, eps2, tol_deg_in_cluster1, tol_deg_out_cluster1, \
                  tol_deg_in_cluster2, tol_deg_out_cluster2, \
                  w1_1, w2_1, w1_2, w2_2, image1, image2, limit1, limit2):
```

```
## Import the images
#denoising
#threshold1 = np.percentile(image1,b1)
image_new1 = cl.denoising2(image1, b1)

#threshold2 = np.percentile(image2,b2)
image_new2 = cl.denoising2(image2, b2)

#convert image type
image_new1 = np.array(image_new1, dtype=np.uint8)
image_new2 = np.array(image_new2, dtype=np.uint8)

#get the clean images
#image_new_clean1 = cl.get_clean_image(image_new1, connectivity1)
#image_new_clean2 = cl.get_clean_image(image_new2, connectivity2)
image_new_clean1 = image_new1
image_new_clean2 = image_new2

## Cluster the imges and detect the fibers
#get the pixel locations
threshold_standoutcluster = 0.1
pixel_position1 = cl.pixel_location(image_new_clean1, threshold_standoutcluster)
pixel_position2 = cl.pixel_location(image_new_clean2, threshold_standoutcluster)

#apply cluster method
#apply k-means



if method == 'KMeans':

    k_instance_kmean1 = \
    KMeans(max_iter=100, n_clusters = n_clusters1, init='k-means++').fit(pixel_position1)
    k_instance_kmean2 = \
    KMeans(max_iter=100, n_clusters = n_clusters2, init='k-means++').fit(pixel_position2)

    cluster_label1 = k_instance_kmean1.labels_
    cluster_label2 = k_instance_kmean2.labels_
    cluster_num1 = k_instance_kmean1.cluster_centers_.shape[0]
    cluster_num2 = k_instance_kmean2.cluster_centers_.shape[0]


if method == 'SpectralClustering':

    spectral_model_rbf1 = SpectralClustering(n_clusters = n_clusters1, affinity ='rbf')
    spectral_model_rbf2 = SpectralClustering(n_clusters = n_clusters2, affinity ='rbf')

    labels_rbf1 = \
    spectral_model_rbf1.fit_predict(pixel_position1)
    labels_rbf2 = \
    spectral_model_rbf2.fit_predict(pixel_position2)

    cluster_label1 = labels_rbf1
    cluster_label2 = labels_rbf2
    cluster_num1 = labels_rbf1.max()
    cluster_num2 = labels_rbf2.max()


if method == "AgglomerativeClustering":

    cluster_agg1 = AgglomerativeClustering(n_clusters = n_clusters1).fit(pixel_position1)
    cluster_agg2 = AgglomerativeClustering(n_clusters = n_clusters2).fit(pixel_position2)

    cluster_label1 = cluster_agg1.labels_
    cluster_label2 = cluster_agg2.labels_
    cluster_num1 = cluster_label1.max()
    cluster_num2 = cluster_label2.max()


if method == "DBSCAN":

    cluster_DBSCAN1 = DBSCAN(eps = eps1, min_samples=n_clusters1).fit(pixel_position1)
    cluster_DBSCAN2 = DBSCAN(eps = eps2, min_samples=n_clusters2).fit(pixel_position2)
```

```
        cluster_label1 = cluster_DBSCAN1.labels_
        cluster_label2 = cluster_DBSCAN2.labels_

        cluster_num1 = cluster_label1.max()
        cluster_num2 = cluster_label2.max()


    #cluster
    cluster_dict1, cluster_num_new1 = cl.pixel_cluster2(cluster_label1, pixel_position1, cluster_num1, limit1)
    cluster_dict2 = cl.pixel_cluster(cluster_label2, pixel_position2, cluster_num2)

    cluster_num1 = cluster_num_new1


    #find the vertices
    right_down_dict1, right_up_dict1, left_down_dict1, left_up_dict1 \
    = cl.cluster_vertex(cluster_num1, cluster_dict1)
    right_down_dict2, right_up_dict2, left_down_dict2, left_up_dict2 \
    = cl.cluster_vertex(cluster_num2, cluster_dict2)

    #find clusters angles
    cluster_angles1 = cl.cluster_angle_fun(cluster_num1, cluster_dict1, \
                                    right_down_dict1, right_up_dict1, \
                                    left_down_dict1, left_up_dict1)
    cluster_angles2 = cl.cluster_angle_fun(cluster_num2, cluster_dict2, \
                                    right_down_dict2, right_up_dict2, \
                                    left_down_dict2, left_up_dict2)

    #find fibers angles
    tol_in_cluster1 = tol_deg_in_cluster1*pi/180
    tol_out_cluster1 = tol_deg_in_cluster1*pi/180
    tol_in_cluster2 = tol_deg_in_cluster2*pi/180
    tol_out_cluster2 = tol_deg_in_cluster2*pi/180


    cluster_dict_new1, right_down_dict_new1, right_up_dict_new1, \
    left_down_dict_new1, left_up_dict_new1 \
    = cl.cluster_update(cluster_num1, cluster_angles1, tol_in_cluster1, tol_out_cluster1, \
                 cluster_dict1, right_down_dict1, right_up_dict1, \
                 left_down_dict1, left_up_dict1)

    cluster_dict_new2, right_down_dict_new2, right_up_dict_new2, \
    left_down_dict_new2, left_up_dict_new2 \
    = cl.cluster_update(cluster_num2, cluster_angles2, tol_in_cluster2, tol_out_cluster2, \
                 cluster_dict2, right_down_dict2, right_up_dict2, \
                 left_down_dict2, left_up_dict2)

    cluster_angle_new1 = cl.cluster_angle_fun(cluster_num1, cluster_dict_new1, \
                                       right_down_dict_new1, right_up_dict_new1, \
                                       left_down_dict_new1, left_up_dict_new1)

    cluster_angle_new2 = cl.cluster_angle_fun(cluster_num2, cluster_dict_new2, \
                                       right_down_dict_new2, right_up_dict_new2, \
                                       left_down_dict_new2, left_up_dict_new2)

    cluster_dict_new_posi1, cluster_dict_new_nega1, right_down_dict_posi1, right_up_dict_posi1, \
    left_down_dict_posi1, left_up_dict_posi1, right_down_dict_nega1, right_up_dict_nega1, \
    left_down_dict_nega1, left_up_dict_nega1\
    = cl.cluster_group(cluster_num1, cluster_dict_new1, cluster_angle_new1, \
        right_down_dict_new1, right_up_dict_new1, \
                left_down_dict_new1, left_up_dict_new1)

    cluster_dict_new_posi2, cluster_dict_new_nega2, right_down_dict_posi2, right_up_dict_posi2, \
    left_down_dict_posi2, left_up_dict_posi2, right_down_dict_nega2, right_up_dict_nega2, \
    left_down_dict_nega2, left_up_dict_nega2 \
    = cl.cluster_group(cluster_num2, cluster_dict_new2, cluster_angle_new2, \
        right_down_dict_new2, right_up_dict_new2, \
                left_down_dict_new2, left_up_dict_new2)




    ## Selelct the muscle fibers

    #define the angle sets that will be used for upper pic
    cluster_dict_new1, right_down_dict1, right_up_dict1, left_down_dict1, left_up_dict1 = \
    cluster_dict_new_nega1, right_down_dict_nega1, right_up_dict_nega1, \
```

```
    left_down_dict_nega1, left_up_dict_nega1

    #define the angle sets that will be used for downner pic
    cluster_dict_new2, right_down_dict2, right_up_dict2, left_down_dict2, left_up_dict2 = \
    cluster_dict_new_posi2, right_down_dict_posi2, right_up_dict_posi2, \
    left_down_dict_posi2, left_up_dict_posi2



    value_dict1 = fiv.Value_Funtion(1, w1_1, w2_1, image_new_clean1, \
                                 cluster_dict_new1, right_down_dict1, right_up_dict1, \
                                 left_down_dict1, left_up_dict1)

    index1, value1 = fiv.select_high_value(value_dict1)


    right_pixel1, left_pixel1 = fiv.find_vertices(index1, right_down_dict1, left_up_dict1)


    value_dict2 = fiv.Value_Funtion(2, w1_2, w2_2, image_new_clean2, \
                                 cluster_dict_new2, right_down_dict2, right_up_dict2, \
                                 left_down_dict2, left_up_dict2)

    index2, value2 = fiv.select_high_value(value_dict2)

    right_pixel2, left_pixel2 = fiv.find_vertices(index2, right_up_dict2, left_down_dict2)


    hor_l, hor_r = np.array([45, 1]), np.array([45, 5])

    #angle selected based on the max value
    angle_max1 = angle_compute(right_pixel1, left_pixel1, hor_r, hor_l)
    angle_max2 = angle_compute(right_pixel2, left_pixel2, hor_r, hor_l)




    return angle_max1, angle_max2





def angle_compute(right_pixel1, left_pixel1, right_pixel2, left_pixel2):
    ## Draw the line
        #determine the angles
        diff_up = right_pixel1 - left_pixel1

        if diff_up[1] == 0:
            diff_up[1] = 0.001
        tg_up = -diff_up[0]/diff_up[1]
        angle_up = np.arctan(tg_up)

        diff_down = right_pixel2 - left_pixel2

        if diff_down[1] == 0:
            diff_down[1] = 0.001
        tg_down = -diff_down[0]/diff_down[1]
        angle_down = np.arctan(tg_down)

        angle_diff = abs(angle_down - angle_up)*180/pi

        return angle_diff







#compute the average angle
def angle_from_values(value_dict, right_down_dict, left_up_dict):

    value = 0
    angle_wighted_vec = 0
```

```
    hor_l, hor_r = np.array([45, 1]), np.array([45, 5])

    for n in value_dict.keys():

        if n in value_dict:
            value = value + value_dict[n]

            right_pixel, left_pixel = fiv.find_vertices(n, right_down_dict, left_up_dict)


            angle = angle_compute(right_pixel, left_pixel, hor_r, hor_l)

            angle_wighted = angle*value_dict[n]

            angle_wighted_vec = angle_wighted_vec + angle_wighted


    angle_average = angle_wighted_vec/(value+0.0000001)

    return angle_average


#compute the average angle with corrected weights
def angle_from_values_cw(value_dict, right_down_dict, left_up_dict, last_angle, sigma):

    value = 0
    angle_wighted_vec = 0

    hor_l, hor_r = np.array([45, 1]), np.array([45, 5])

    for n in value_dict.keys():

        if n in value_dict:

            right_pixel, left_pixel = fiv.find_vertices(n, right_down_dict, left_up_dict)


            angle = angle_compute(right_pixel, left_pixel, hor_r, hor_l)

            #curve the weights
            r = curve_rate(angle, last_angle, sigma)

            #print('r: % 2f' %r)

            value_curve = r * value_dict[n]
            value = value + value_curve

            angle_wighted = angle * value_curve

            angle_wighted_vec = angle_wighted_vec + angle_wighted

    angle_average = angle_wighted_vec/(value + 0.00000001)

    return angle_average

#Compute the curve_rate
def curve_rate(angle, last_angle, sigma):
    core = -math.pow((angle - last_angle)/(last_angle + 0.00000000001), 2) / sigma / sigma
    #print('core: % 2f' %core)
    core = max(core, -100000)
    #print('core_after: % 2f' %core)
    r = math.exp(core)
    return r

##Define Image Augmentation Functions
#Define distance
def my_dist(x1,y1,x2,y2):
    dist = math.sqrt((x2 - x1)**2 + (y2 - y1)**2)
    return dist



#Create the ellipse field
def ellipse_augmentation3(image, c1, c2, alpha1, alpha2, alpha3, m1, m2, m3):
    height = image.shape[0]
    width = image.shape[1]
```

```
    image_augment = np.zeros((height,width), np.uint8)

    c2c = my_dist(c1[0],c1[1],c2[0],c2[1])


    a_two1 = abs(1+alpha1)*c2c
    a_two2 = abs(1+alpha2)*c2c
    a_two3 = abs(1+alpha3)*c2c

        #exception
    if a_two2 < a_two1:
        a_two2 = a_two1
    if a_two3 < a_two2:
        a_two3 = a_two2


    for i in range(height):
        for j in range (width):

            L = my_dist(i, j, c1[0], c1[1]) + my_dist(i, j, c2[0], c2[1])
            if L < a_two1:
                image_augment[i,j] = image[i,j]*m1*0.25
            elif L >= a_two1 and L < a_two2:
                image_augment[i,j] = image[i,j]*m2*0.25
            elif L >= a_two2 and L < a_two3:
                image_augment[i,j] = image[i,j]*m3*0.25
            else:
                image_augment[i,j] = image[i,j]*0.25


    return image_augment

#Create the ellipse field
def ellipse_augmentation4(image, c1, c2, alpha1, alpha2, alpha3, m1, m2, m3):
    height = image.shape[0]
    width = image.shape[1]
    image_augment = np.zeros((height,width), np.uint8)

    #c11 = max(int(c1[1]*1.2), 509)
    #c22 = max(int(c2[1]*0.8), 1)

    c2c = my_dist(c1[0],int(c1[1]*1.15),c2[0],int(c2[1]*0.85))


    a_two1 = abs(1+alpha1)*c2c
    a_two2 = abs(1+alpha2)*c2c
    a_two3 = abs(1+alpha3)*c2c

        #exception
    if a_two2 < a_two1:
        a_two2 = a_two1
    if a_two3 < a_two2:
        a_two3 = a_two2


    for i in range(height):
        for j in range (width):

            L = my_dist(i, j, c1[0], c1[1]) + my_dist(i, j, c2[0], c2[1])
            if L < a_two1:
                image_augment[i,j] = image[i,j]*m1*0.25
            elif L >= a_two1 and L < a_two2:
                image_augment[i,j] = image[i,j]*m2*0.25
            elif L >= a_two2 and L < a_two3:
                image_augment[i,j] = image[i,j]*m3*0.25
            else:
                image_augment[i,j] = image[i,j]*0.25


    return image_augment


#Create the ellipse field
def ellipse_augmentation5(image, c1, c2, alpha1, alpha2, alpha3, m1, m2, m3):
    height = image.shape[0]
    width = image.shape[1]
    image_augment = np.zeros((height,width), np.uint8)
```

```
    c11 = int(c1[1]*1.2)
    c22 = int(c2[1]*0.8)

    if c11 - c22 < 300:
        c11 = 500
        c22 = 80

    c2c = my_dist(c1[0],c11,c2[0],c22)


    a_two1 = abs(1+alpha1)*c2c
    a_two2 = abs(1+alpha2)*c2c
    a_two3 = abs(1+alpha3)*c2c

        #exception
    if a_two2 < a_two1:
        a_two2 = a_two1
    if a_two3 < a_two2:
        a_two3 = a_two2


    for i in range(height):
        for j in range (width):

            L = my_dist(i, j, c1[0], c1[1]) + my_dist(i, j, c2[0], c2[1])
            if L < a_two1:
                image_augment[i,j] = image[i,j]*m1*0.25
            elif L >= a_two1 and L < a_two2:
                image_augment[i,j] = image[i,j]*m2*0.25
            elif L >= a_two2 and L < a_two3:
                image_augment[i,j] = image[i,j]*m3*0.25
            else:
                image_augment[i,j] = image[i,j]*0.25


    return image_augment

#Create the ellipse field
def ellipse_augmentation6(image, c1, c2, alpha1, alpha2, alpha3, m1, m2, m3):
    height = image.shape[0]
    width = image.shape[1]
    image_augment = np.zeros((height,width), np.uint8)

    c11 = int(c1[1]*1.2)
    c22 = int(c2[1]*0.8)

    if c11 - c22 < 200:
        c11 = max(int(c1[1] + 100), 509)
        c22 = max(int(c2[1] - 100), 1)

    c2c = my_dist(c1[0],c11,c2[0],c22)


    a_two1 = abs(1+alpha1)*c2c
    a_two2 = abs(1+alpha2)*c2c
    a_two3 = abs(1+alpha3)*c2c

        #exception
    if a_two2 < a_two1:
        a_two2 = a_two1
    if a_two3 < a_two2:
        a_two3 = a_two2


    for i in range(height):
        for j in range (width):

            L = my_dist(i, j, c1[0], c1[1]) + my_dist(i, j, c2[0], c2[1])
            if L < a_two1:
                image_augment[i,j] = image[i,j]*m1*0.25
            elif L >= a_two1 and L < a_two2:
                image_augment[i,j] = image[i,j]*m2*0.25
            elif L >= a_two2 and L < a_two3:
                image_augment[i,j] = image[i,j]*m3*0.25
            else:
                image_augment[i,j] = image[i,j]*0.25
```

```
    return image_augment



#Create the ellipse field
#Create the ellipse field
def ellipse_augmentation7(image, c1, c2, alpha1, alpha2, alpha3, m1, m2, m3):
    height = image.shape[0]
    width = image.shape[1]
    image_augment = np.zeros((height,width), np.uint8)


    c2c = my_dist(c1[0],c1[1],c2[0],c2[1])


    a_two1 = abs(1+alpha1)*c2c
    a_two2 = abs(1+alpha2)*c2c
    a_two3 = abs(1+alpha3)*c2c

        #exception
    if a_two2 < a_two1:
        a_two2 = a_two1
    if a_two3 < a_two2:
        a_two3 = a_two2


    for i in range(height):
        for j in range (width):

            L = my_dist(i, j, c1[0], c1[1]) + my_dist(i, j, c2[0], c2[1])
            if L < a_two1:
                image_augment[i,j] = image[i,j]*m1*0.25
            elif L >= a_two1 and L < a_two2:
                image_augment[i,j] = image[i,j]*m2*0.25
            elif L >= a_two2 and L < a_two3:
                image_augment[i,j] = image[i,j]*m3*0.25
            else:
                image_augment[i,j] = image[i,j]*0.25


    return image_augment


def f1(t_i, hr, hl, sigmar, sigmal):
    if t_i < -hl:
        r = np.exp(-((t_i + hl)/sigmal)*((t_i + hl)/sigmal) / 2)
    elif t_i > hr:
        r = np.exp(-((t_i - hr)/sigmar)*((t_i - hr)/sigmar) / 2)
    else:
        r = 1
    return r


def viscosity_process(angle, last_angle):
    ratio = (angle - last_angle)/last_angle
    r = f1(ratio, 0.05, 0.025, 0.05, 0.024)
    final_angle = (1-r) * last_angle + r * angle
    return final_angle


def viscosity_weight(angle, last_angle):
    ratio = (angle - last_angle)/last_angle
    r = f1(ratio, 0.02, 0.0025, 0.05, 0.024)
    return r



#create feasible region surrounding the last detected fibers
def feasible_region(right_pixel, left_pixel, image, radius):

    height = image.shape[0]
    width = image.shape[1]
    #create a new image
    #image_region = np.zeros((height,width,3), np.uint8)
    #image_region.fill(255)
```

```
    image_region = np.zeros((height,width), np.uint8)



        #line properties
    if right_pixel[0] - left_pixel[0] != 0:

        k = (right_pixel[1] - left_pixel[1])/(right_pixel[0] - left_pixel[0])
        b = left_pixel[1] - left_pixel[0] * k


        #positive direction
        for i in range(height):

            #new points along the line
            hight_loco = i
            width_loco = k*hight_loco + b

            #make sure the points are on the image
            if width_loco > 0:
                #create region for this point
                if width_loco - radius <= 0:
                    lower_bar = 0
                else:
                    lower_bar = width_loco - radius

                if width_loco + radius >= width:
                    upper_bar = width
                else:
                    upper_bar = width_loco + radius

                for j in range(int(lower_bar), int(upper_bar)):

                    image_region[i,j] = image[i,j]


    else:
        #slope is 0
        for j in range(width):
            if left_pixel[1] - radius <= 0:
                lower_bar = 0
            else:
                lower_bar = left_pixel[1] - radius

            if left_pixel[1] + radius >= height:
                upper_bar = height
            else:
                upper_bar = left_pixel[1] + radius

            for i in range(int(lower_bar), int(upper_bar)):
                image_region[i,j] = image[i,j]






    return image_region





##Define Reference Extract Functions
#extract the red label
def extract_red(image):
    image_r = np.zeros((image.shape[0],image.shape[1],3), np.uint8)

    for i in range(image.shape[0]):
        for j in range(image.shape[1]):
            b,g,r = image[i,j]
            if((r-b)>40 and (r-g)>40):
                b=0
                g=0
                r=0

            else:
                b=255
```

```
                g=255
                r=255

            image_r[i,j]=[r,g,b]

    return image_r
#extract the green label
def extract_green(image):
    image_g = np.zeros((image.shape[0],image.shape[1],3), np.uint8)

    for i in range(image.shape[0]):
        for j in range(image.shape[1]):
            b,g,r = image[i,j]
            if(g-b>40 and g-r>40):
                b=0
                g=0
                r=0

            else:
                b=255
                g=255
                r=255

            image_g[i,j]=[r,g,b]

    return image_g

#create value field (1 layers)
def value_field_1(image, radius):

    height = image.shape[0]
    width = image.shape[1]

    image_ref = np.zeros((height,width,3), np.uint8)
    image_ref.fill(255)

    for i in range(height):
        for j in range(width):

            b,g,r = image[i,j]

            if b < 0.1 and g < 0.1 and r < 0.1:

                up = i-radius
                down = i+radius
                left = j-radius
                right = j+radius

                if up < 0:
                    up = 1
                if down > height-1:
                    down = height-1
                if left < 0:
                    left = 1
                if right > width-1:
                    right = width-1

                for n in range(up, down):
                    for m in range(left, right):

                        image_ref[n,m] = [0,0,0]
    return image_ref


#create value field (3 layers)
#colors of the three layers are [90,90,90], [50,50,50], [0,0,0]
#the arguments r1 r2 r3 in value_field(image_l_red, r1, r2, r3) are the diameters of pixles, which defines the size

def value_field_3(image, r1, r2, r3):

    height = image.shape[0]
    width = image.shape[1]

    image_field = np.zeros((height,width,3), np.uint8)
    image_field.fill(255)

    #exception
```

```
    if r2 < r1:
        r2 = r1
    if r3 < r2:
        r3 = r2

    #fill outter layer
    for i in range(height):
        for j in range(width):

            b,g,r = image[i,j]
            if b < 0.1 and g < 0.1 and r < 0.1:

                up = i-r3
                down = i+r3
                left = j-r3
                right = j+r3

                if up < 0:
                    up = 1
                if down > height-1:
                    down = height-1
                if left < 0:
                    left = 1
                if right > width-1:
                    right = width-1

                for n in range(up, down):
                    for m in range(left, right):
                        image_field[n,m] = [90,90,90]

    #fill middle layer
    for i in range(height):
        for j in range(width):

            b,g,r = image[i,j]
            if b < 0.1 and g < 0.1 and r < 0.1:

                up = i-r2
                down = i+r2
                left = j-r2
                right = j+r2

                if up < 0:
                    up = 1
                if down > height-1:
                    down = height-1
                if left < 0:
                    left = 1
                if right > width-1:
                    right = width-1

                for n in range(up, down):
                    for m in range(left, right):
                        image_field[n,m] = [50,50,50]

    #fill outter layer
    for i in range(height):
        for j in range(width):

            b,g,r = image[i,j]
            if b < 0.1 and g < 0.1 and r < 0.1:

                up = i-r1
                down = i+r1
                left = j-r1
                right = j+r1

                if up < 0:
                    up = 1
                if down > height-1:
                    down = height-1
                if left < 0:
                    left = 1
                if right > width-1:
                    right = width-1

                for n in range(up, down):
```

```
                for m in range(left, right):
                    image_field[n,m] = [0,0,0]


    return image_field
```

```
__author__ = 'bao'

import cv2
import numpy as np
from numpy import *
import pandas as pd
import skimage



#erase the pixles whose values are less than treshold
def denoising(image, threshold):
    image_new = np.zeros(image.shape)
    for x in range(0, image.shape[0]):
        for y in range(0, image.shape[1]):
            if image[x, y] >= threshold:
                image_new[x, y] = image[x, y]
    return image_new


#erase the pixles whose values are less than an intensity
def denoising2(image, intensity):
    image_new = np.zeros(image.shape)
    for x in range(0, image.shape[0]):
        for y in range(0, image.shape[1]):
            if image[x, y] >= intensity:
                image_new[x, y] = image[x, y]
    return image_new


#erase small connected components
def get_clean_image(image_new, connectivity_setting):

    nb_components, output, stats, centroids = cv2.connectedComponentsWithStats(image_new, \
                                                                                 connectivity = connectivity_setting)
    sizes = stats[1:, -1]
    size_threshold = np.percentile(sizes,60) # lower % will be removed
    image_new_clean = image_new
    for i in range(0, nb_components - 1):
        if sizes[i] <= size_threshold:
            image_new_clean[output == i + 1] = 0
    return image_new_clean


#function for extracting the position of the bright pixels in the coordinate
def pixel_location(image, threshold):
    pixel_position = []
    for x in range(0, image.shape[0]):
        for y in range(0, image.shape[1]):
            if image[x, y] >= threshold:
                pixel_position.append([x, y])
    pixel_position = array(pixel_position)
    return pixel_position


#assign the pixel to its corresponding cluster
def pixel_cluster(cluster_label, pixel_position, cluster_num):
    cluster_dict = {}
    for n in range(0, cluster_num):
        dummy = cluster_label == n
        pixel_labeled = pixel_position[dummy]
        cluster_dict.update( {n : pixel_labeled} )
    return cluster_dict


def pixel_cluster2(cluster_label, pixel_position, cluster_num, limit):
    cluster_dict = {}
    cluster_num_new = 0
    for n in range(0, cluster_num):
        dymmy = cluster_label == n
        pixel = pixel_position[dymmy]


        pixel_ver = np.zeros((pixel.shape[0], 1))
        pixel_hor = np.zeros((pixel.shape[0], 1))
```

```
        for i in range(0, pixel.shape[0]):
            pixel_ver[i] = pixel[i][0]
            pixel_hor[i] = pixel[i][1]

        center = np.take(pixel_hor, pixel_hor.size // 2)
        firstQ = np.take(pixel_hor, pixel_hor.size // 4)
        thirdQ = np.take(pixel_hor, pixel_hor.size // 1.33)

        rightest = np.amax(pixel_hor, axis=0)
        leftest = np.amin(pixel_hor, axis=0)



        location_center = np.where(pixel_hor == center)
        location_firstQ = np.where(pixel_hor == firstQ)
        location_thirdQ = np.where(pixel_hor == thirdQ)


        pixel_ver_center_temp = np.zeros((location_center[0].shape[0],1))
        pixel_ver_firstQ_temp = np.zeros((location_firstQ[0].shape[0],1))
        pixel_ver_thirdQ_temp = np.zeros((location_thirdQ[0].shape[0],1))

        for i in range(0, location_center[0].shape[0]):
            pixel_ver_center_temp[i] = pixel_ver[location_center[0][i]]
        for i in range(0, location_firstQ[0].shape[0]):
            pixel_ver_firstQ_temp[i] = pixel_ver[location_firstQ[0][i]]
        for i in range(0, location_thirdQ[0].shape[0]):
            pixel_ver_thirdQ_temp[i] = pixel_ver[location_thirdQ[0][i]]

        d_center = np.amax(pixel_ver_center_temp)
        u_center = np.amin(pixel_ver_center_temp)
        d_firstQ = np.amax(pixel_ver_firstQ_temp)
        u_firstQ = np.amin(pixel_ver_firstQ_temp)
        d_thirdQ = np.amax(pixel_ver_thirdQ_temp)
        u_thirdQ = np.amin(pixel_ver_thirdQ_temp)



        if int(d_center - u_center) < limit and int(d_firstQ - u_firstQ) < limit and int(d_thirdQ - u_thirdQ) < lim


            cluster_dict.update( {n : pixel} )
            cluster_num_new = cluster_num_new + 1


    return cluster_dict, cluster_num_new




#find the corners of a cluster  (right or left first, then down or up)
def cluster_vertex(cluster_num, cluster_dict):
    right_down_dict = {}
    right_up_dict = {}
    left_down_dict = {}
    left_up_dict = {}

    for n in range(0, cluster_num):
        if n in cluster_dict:
            pixel = cluster_dict[n]
            pixel_ver = np.zeros((pixel.shape[0], 1))
            pixel_hor = np.zeros((pixel.shape[0], 1))

            for i in range(0, pixel.shape[0]):
                pixel_ver[i] = pixel[i][0]
                pixel_hor[i] = pixel[i][1]

            rightest = np.amax(pixel_hor, axis=0)
            leftest = np.amin(pixel_hor, axis=0)

            location_right = np.where(pixel_hor == rightest)
            location_left = np.where(pixel_hor == leftest)

            pixel_ver_right_temp = np.zeros((location_right[0].shape[0],1))
            pixel_ver_left_temp = np.zeros((location_left[0].shape[0],1))

            for i in range(0, location_right[0].shape[0]):
```

```
                pixel_ver_right_temp[i] = pixel_ver[location_right[0][i]]
            for i in range(0, location_left[0].shape[0]):
                pixel_ver_left_temp[i] = pixel_ver[location_left[0][i]]

            d_r = np.amax(pixel_ver_right_temp, axis=0)
            d_l = np.amax(pixel_ver_left_temp, axis=0)
            u_r = np.amin(pixel_ver_right_temp, axis=0)
            u_l = np.amin(pixel_ver_left_temp, axis=0)

            right_down = array([d_r[0], rightest[0]])
            left_down = array([d_l[0], leftest[0]])
            right_up = array([u_r[0], rightest[0]])
            left_up = array([u_l[0], leftest[0]])

            right_down_dict.update( {n : right_down} )
            right_up_dict.update( {n : right_up} )
            left_down_dict.update( {n : left_down} )
            left_up_dict.update( {n : left_up} )

    return right_down_dict, right_up_dict, left_down_dict, left_up_dict




# find the orientations of the clusters
def cluster_angle_fun(cluster_num, cluster_dict, right_down_dict, right_up_dict, left_down_dict, left_up_dict):
    cluster_angle_dict = {}
    for n in range(0, cluster_num):
        if n in cluster_dict:

            diff1 = right_down_dict[n] - left_up_dict[n]
            if diff1[1] == 0:
                diff1[1] = 0.001
            tg1 = -diff1[0]/diff1[1]
            angle1 = np.arctan(tg1)

            diff2 = right_up_dict[n] - left_down_dict[n]
            if diff2[1] == 0:
                diff2[1] = 0.001
            tg2 = -diff2[0]/diff2[1]
            angle2 = np.arctan(tg2)

            angle = 0.5*(angle1 + angle2)

            cluster_angle_dict.update( {n : angle} )

    return cluster_angle_dict




#update clusters ---- splice the clusters which are actually belonged to the same muscle filbers
def cluster_update(cluster_num, cluster_angles, tol1, tol2, cluster_dict, \
                   right_down_dict, right_up_dict, left_down_dict, left_up_dict):

    right_down_dict_new = {}
    right_up_dict_new = {}
    left_down_dict_new = {}
    left_up_dict_new = {}

    cluster_dict_new = {}

    used = set()

    for i in range(0, cluster_num):
        #print(used)
        #print("i : % 2d" % i)
        if i not in used:

            #print("i : % 2d" % i)
            used.add(i)
            cluster_dict_new.update( {i : cluster_dict[i]} )

            #print('cluster i: % 2d' % i)
            #print(cluster_dict_new[i].shape[0])
```

```
            right_down_dict_new.update( {i : right_down_dict[i]} )
            right_up_dict_new.update( {i : right_up_dict[i]} )
            left_down_dict_new.update( {i : left_down_dict[i]} )
            left_up_dict_new.update( {i : left_up_dict[i]} )

            #print(i)

            for j in range(i + 1, cluster_num):
                if j not in used:
                    diff_angle1 = cluster_angles[i] - cluster_angles[j]
                    if abs(diff_angle1) < tol1:

                        #more robust if we change to down or up first
                        rightesti = np.amax([right_down_dict[i][1], right_up_dict[i][1]], axis=0)
                        rightestj = np.amax([right_down_dict[j][1], right_up_dict[j][1]], axis=0)
                        leftesti = np.amin([left_down_dict[i][1], left_up_dict[i][1]], axis=0)
                        leftestj = np.amin([left_down_dict[j][1], left_up_dict[j][1]], axis=0)


                        pair_compare = (leftesti, rightesti, leftestj, rightestj)
                        vertex_combo = (right_down_dict[i], right_up_dict[i], left_down_dict[i], left_up_dict[i], \
                                        right_down_dict[j], right_up_dict[j], left_down_dict[j], left_up_dict[j])

                        if rightesti - rightestj < 0:
                            pair_compare = (leftestj, rightestj, leftesti, rightesti)
                            vertex_combo = (right_down_dict[j], right_up_dict[j], left_down_dict[j], left_up_dict[j
                                            right_down_dict[i], right_up_dict[i], left_down_dict[i], left_up_dict[i

                        if pair_compare[0] - pair_compare[3] > 0:

                            diff = 0.5 * (vertex_combo[6] + vertex_combo[7] - vertex_combo[0] - vertex_combo[1])
                            if diff[1] == 0:
                                diff[1] = 0.001
                            diff_angle2 = np.arctan(-diff[0]/diff[1])

                            if abs(diff_angle2 - 0.5 * (cluster_angles[i] + cluster_angles[j])) < tol2:
                                #print("j : % 2d" % j)
                                used.add(j)
                                cluster_dict_new.update( {i : np.concatenate((cluster_dict_new[i], cluster_dict[j])

                                #print('updated cluster i: % 2d' % i)
                                #print(cluster_dict_new[i].shape[0])

                                if right_down_dict[j][1] > right_down_dict[i][1]:
                                    right_down_dict_new.update( {i : right_down_dict[j]} )
                                    right_down_dict[i][1] = right_down_dict[j][1]
                                if right_up_dict[j][1] > right_up_dict[i][1]:
                                    right_up_dict_new.update( {i : right_up_dict[j]} )
                                    right_up_dict[i][1] = right_up_dict[j][1]
                                if left_down_dict[j][1] < left_down_dict[i][1]:
                                    left_down_dict_new.update( {i : left_down_dict[j]} )
                                    left_down_dict[i][1] = left_down_dict[j][1]
                                if left_up_dict[j][1] < left_up_dict[i][1]:
                                    left_up_dict_new.update( {i : left_up_dict[j]} )
                                    left_up_dict[i][1] = left_up_dict[j][1]



            #cluster_dict_new.update( {i : cluster_dict[i]} )


    return cluster_dict_new, right_down_dict_new, right_up_dict_new, left_down_dict_new, left_up_dict_new




#devide the cluster to two groups, one has position angles and the other has negative
def cluster_group(cluster_num, cluster_dict, cluster_angle, \
                  right_down_dict, right_up_dict, left_down_dict, left_up_dict):
    cluster_dict_posi = {}
    cluster_dict_nega = {}

    right_down_dict_posi = {}
    right_up_dict_posi = {}
    left_down_dict_posi = {}
    left_up_dict_posi = {}
```

```
    right_down_dict_nega = {}
    right_up_dict_nega = {}
    left_down_dict_nega = {}
    left_up_dict_nega = {}

    for n in range(0, cluster_num):
        if n in cluster_dict:
            if cluster_angle[n] > 0:
                cluster_dict_posi.update( {n : cluster_dict[n]} )

                right_down_dict_posi.update( {n : right_down_dict[n]} )
                right_up_dict_posi.update( {n :  right_up_dict[n]} )
                left_down_dict_posi.update( {n : left_down_dict[n]} )
                left_up_dict_posi.update( {n : left_up_dict[n]} )

            else:
                cluster_dict_nega.update( {n : cluster_dict[n]} )

                right_down_dict_nega.update( {n : right_down_dict[n]} )
                right_up_dict_nega.update( {n :  right_up_dict[n]} )
                left_down_dict_nega.update( {n : left_down_dict[n]} )
                left_up_dict_nega.update( {n : left_up_dict[n]} )

    return cluster_dict_posi, cluster_dict_nega, right_down_dict_posi, right_up_dict_posi, \
left_down_dict_posi, left_up_dict_posi, right_down_dict_nega, right_up_dict_nega, \
left_down_dict_nega, left_up_dict_nega
```

Fiber_Value

```
__author__ = 'bao'


#define value function
def Value_Funtion(who, w1, w2, image_new_clean, cluster_dict, right_down_dict, right_up_dict, left_down_dict, left_
    value_dict = {}

    value = 0
    brightness = 0

    for i in cluster_dict.keys():
        for j in range (0, len(cluster_dict[i])):

            x = cluster_dict[i][j][0]
            y = cluster_dict[i][j][1]

            brightness = brightness + image_new_clean[x][y]

        value = brightness/(j+1) + w1 * brightness + w2 * abs(right_down_dict[i][1] + right_up_dict[i][1] - left_dc

        if who == 1 and right_down_dict[i][0] + right_up_dict[i][0] - left_down_dict[i][0] - left_up_dict[i][0] < 0
            value = 0

        value_dict.update({i: value})
        brightness = 0
        value = 0

    return value_dict



#select the cluster whos has the highest value
def select_high_value(value_dict):
    #print('func is called')

    index = 0
    value = 0

    for n in value_dict.keys():
        #print('cluster #', n)
        if n in value_dict:
            #print('cluster # in dict', n)
            #print('current value', value)
            if value < value_dict[n]:
                value = value_dict[n]
                index = n
    #print('updated value', value)
    #print('selected cluster', index)
    return index, value




#find the vertex to plot lines upper one
def find_vertices(index, right, left):

    right_pixel = right[index]
    left_pixel = left[index]


    return right_pixel, left_pixel
```

How to Design Parameters

This code has a lot of parameters, e.g., ROI, denoising threshold, ellipse property, clustering algorithm, reclustering angles, value function weights, etc. To obtain satisfactory results, we need to select good parameters for the specific data. This document will show some tricks for tuning the parameters to get good results.

1. Open Jupyter notebook
   We will use Jupyter notebook to show the example.

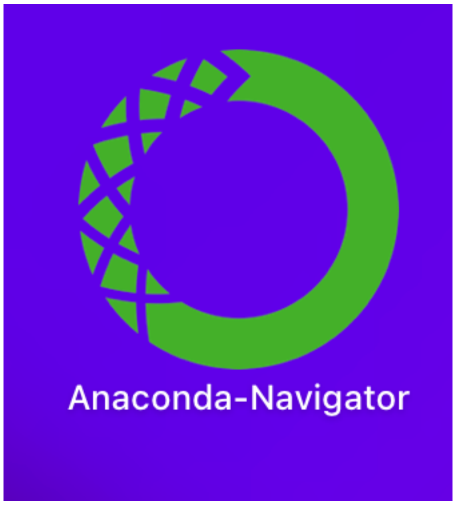

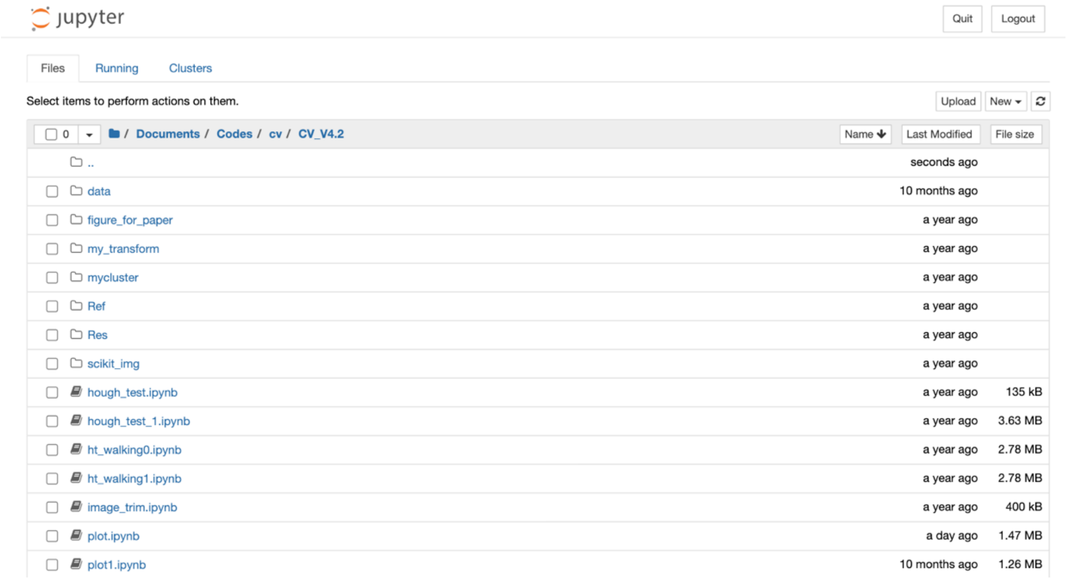

2. Import modules

```
In [1]: #import standard modules
import math
import time
import numpy as np
from numpy import *
import pandas as pd
import matplotlib.pyplot as plt
from pylab import *
from PIL import Image

import skimage
from sklearn.cluster import KMeans
from sklearn.cluster import SpectralClustering
from sklearn.cluster import AgglomerativeClustering
from sklearn.cluster import DBSCAN

from sklearn.decomposition import PCA
from sklearn.metrics import silhouette_score


import cv2

#import my modules
import Fiber_Select as fs
```

3. Define functions

Define all the functions shown in the code. For example:

Define Necessary Functions

```
In [2]: def show_cluster(image, cluster):
    image_cluster = np.zeros((image.shape[0], image.shape[1]), np.uint8)
    image_cluster.fill(0)
    for n in range(cluster.shape[0]):
        height = cluster[n][0]
        width = cluster[n][1]
        image_cluster[height, width] = image[height, width]
    return image_cluster


#Define distance
def my_dist(x1,y1,x2,y2):
    dist = math.sqrt((x2 - x1)**2 + (y2 - y1)**2)
    return dist

def cut_image_a(image):

    #trim the images
    image = image[161:468, 114:791]

    return image


def cut_image_o2(image):

    #trim the images
    image = image[200:450, 120:790]

    return image

#Create the ellipse field
def ellipse_augmentation3(image, c1, c2, alpha1, alpha2, alpha3, m1, m2, m3):
    height = image.shape[0]
    width = image.shape[1]
    image_augment = np.zeros((height,width), np.uint8)

    c2c = my_dist(c1[0],c1[1],c2[0],c2[1])
```

We can just put the codes in the cell and run them or directly import them.

4. Split the image to two parts: up and down

4.1 Let find where to cut first.

Find cut position

In [6]:
```python
number = 31

image = cv2.imread('./data/Ankle_A02/angle_15_trial1/Original/TA' + str(number) + '.tif', cv2.IMREAD_GRAYSCALE)
#image = cv2.imread('./data/unknown/original_images/TA' + str(number) + '.tif', cv2.IMREAD_GRAYSCALE)
plt.imshow(image)

offset_A02a15t10 = 0


image_t = trim_image_A02a15t10(image)
image_t_t= image_t.astype(np.uint8)
image_zoom = image_t_t[45:115, 350:450]
threshold = np.percentile(image_zoom, 85)
image_zoom_clean = denoising(image_zoom, threshold)
image_zoom_clean = np.array(image_zoom_clean, dtype=np.uint8)
cut_position = find_cut_position(image_zoom_clean) + offset_A01a5t10

plt.imshow(image_zoom_clean)

print('cut_position: %d' %(cut_position))
```

cut_position: 28

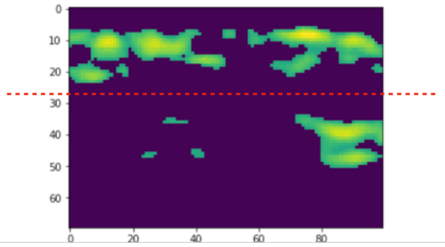

```python
def trim_image_A02a10t10(image):

    #trim the images
    image = image[95:580, 220:690]

    return image
```

```python
def find_cut_position(image_zoom_clean):
    row = image_zoom_clean.shape[0]
    col = image_zoom_clean.shape[1]

    temp_array = []

    for i in range(1, row):
        for j in range (1, col):
            if image_zoom_clean[i][j] > 0:
                temp_array.append(i)
                break
    cut_position = int((min(temp_array) + max(temp_array))/2)
    return cut_position
```

4.2 Then, we can cut.

In [10]:
```python
image = cv2.imread('./data/Ankle_A02/angle_15_trial1/Original/TA' + str(number) + '.tif', cv2.IMREAD_GRAYSCALE)
#image = cv2.imread('./data/unknown/original_images/TA' + str(number) + '.tif', cv2.IMREAD_GRAYSCALE)

plt.imshow(image)

image_o_t = trim_image_A02a15t10(image)

plt.imshow(image_o_t)

trim_hor_start_A02a15t10 = 20
trim_ver_A02a15t10 = 190
trim_hor_A02a15t10 = 450
basic_cut_A02a15t10 = 60

image1, image2 = cut_image_general(image_o_t, trim_ver_A02a15t10, trim_hor_start_A02a15t10, \
                                   trim_hor_A02a15t10, basic_cut_A02a15t10, cut_position)


plt.figure(figsize = (12, 12))
plt.imshow(image1)
plt.figure(figsize = (12, 12))
plt.imshow(image2)
```

Out[10]: <matplotlib.image.AxesImage at 0x7f8fc8bd8790>

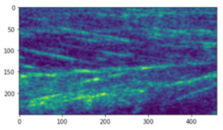

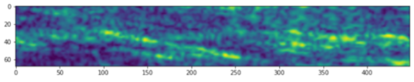

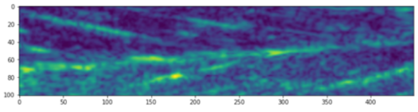

5. Augment the region that may contain the interested muscles
   We take the upper half as an example.

Augment the Image

In [12]:
```python
right_pixel1_last, left_pixel1_last, right_pixel2_last, left_pixel2_last \
= np.array([65, 390]), np.array([21, 1]), np.array([50, 1]), np.array([20, 509])

image_augment1 = ellipse_augmentation7(image1, right_pixel1_last, left_pixel1_last, \
                                       0.00250, 0.0075, 0.00925, 3.75, 3.25, 2.00)

image_augment2 = ellipse_augmentation7(image2, right_pixel2_last, left_pixel2_last, \
                                       0.000550, 0.001, 0.0075, 2.75, 2.5, 1.75)


plt.figure(figsize = (12, 12))
plt.imshow(image_augment1)
```

Out[12]:
```
<matplotlib.image.AxesImage at 0x7f8fe885c910>
```

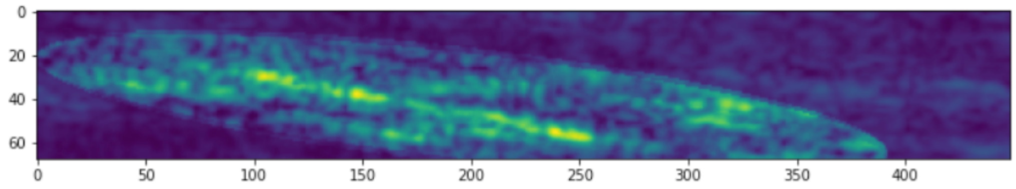

6. If ellipse augmentation is not necessary for some images, we can turn it off:

No Augment

In [13]:
```python
# optional
image_augment1 = image1
image_augment2 = image2
```

7. Set some parameters.
   If they don't work, we can tune them.

In [14]:
```python
b1 = 96 #delete the pixels whos brightness are lower than this percetage for image1
b2 = 92 #delete the pixels whos brightness are lower than this percetage for image2

connectivity1 = 4 #connectivity for cv2.connectedComponentsWithStats for image1
connectivity2 = 4 #connectivity for cv2.connectedComponentsWithStats for image2

n_clusters1 = 40 #number of cluster to be grouped for image1
n_clusters2 = 10 #number of cluster to be grouped for image2

tol_deg_in_cluster1 = 2.75 #degree for checking if two clusters are paralell for image1
tol_deg_out_cluster1 = 2.75 #degree for checking if two clusters are alined for image1
tol_deg_in_cluster2 = 5 #degree for checking if two clusters are paralell for image2
tol_deg_out_cluster2 = 5 #degree for checking if two clusters are alined for image2

w1_1 = 0.2 #weight on total brightness for image1
w2_1 = 50 #weight on fiber length for image1

w1_2 = 0.2 #weight on total brightness for image2
w2_2 = 200 #weight on fiber length for image2


eps1 = 4.5
eps2 = 5.5
mini1 = 20
mini2 = 10
```

8. Denoising

Here, we use the original US image without ellipse augmentation as an example.

Clean the Images

In [15]:
```
#threshold1 = np.percentile(image_augment1, b1)
#image_new1 = denoising(image_augment1, threshold1)
image_new1 = denoising2(image_augment1, 45)

#threshold2 = np.percentile(image_augment2,b2)
#image_new2 = denoising(image_augment2, threshold2)
image_new2 = denoising2(image_augment2, 90)


#convert image type
image_new1 = np.array(image_new1, dtype=np.uint8)
image_new2 = np.array(image_new2, dtype=np.uint8)

#cut the image vertically
#image_new_cutted1 = cut_vertical(image_new1, 28)
image_new_cutted1 = image_new1
#image_new_cutted2 = cut_vertical(image_new2, 50)
image_new_cutted2 = image_new2
#get the clean images

#image_new_clean1 = get_clean_image(image_new_cutted1, connectivity1)
#image_new_clean2 = get_clean_image(image_new_cutted2, connectivity2)
image_new_clean1 = image_new_cutted1
image_new_clean2 = image_new_cutted2



plt.figure(figsize = (12, 12))
plt.imshow(image_new_clean1)
plt.figure(figsize = (12, 12))
plt.imshow(image_new_clean2)
```

Out[15]: <matplotlib.image.AxesImage at 0x7f8fe8463110>

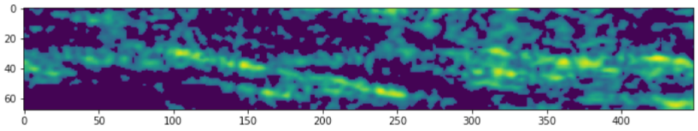

Clean the Images

In [15]:
```
#threshold1 = np.percentile(image_augment1, b1)
#image_new1 = denoising(image_augment1, threshold1)
image_new1 = denoising2(image_augment1, 45)

#threshold2 = np.percentile(image_augment2,b2)
#image_new2 = denoising(image_augment2, threshold2)
image_new2 = denoising2(image_augment2, 90)


#convert image type
image_new1 = np.array(image_new1, dtype=np.uint8)
image_new2 = np.array(image_new2, dtype=np.uint8)

#cut the image vertically
#image_new_cutted1 = cut_vertical(image_new1, 28)
image_new_cutted1 = image_new1
#image_new_cutted2 = cut_vertical(image_new2, 50)
image_new_cutted2 = image_new2
#get the clean images

#image_new_clean1 = get_clean_image(image_new_cutted1, connectivity1)
#image_new_clean2 = get_clean_image(image_new_cutted2, connectivity2)
image_new_clean1 = image_new_cutted1
image_new_clean2 = image_new_cutted2



plt.figure(figsize = (12, 12))
plt.imshow(image_new_clean1)
plt.figure(figsize = (12, 12))
plt.imshow(image_new_clean2)
```


We can tune this number to get a desired clean image.

Pixels with an intensity lower than this, will be removed.

9. Detection

We can perform the following code to get the clusters.

```
In [16]: #get the pixel locations
threshold_standoutcluster = 0.1
pixel_position1 = pixel_location(image_new_clean1, threshold_standoutcluster)
pixel_position2 = pixel_location(image_new_clean2, threshold_standoutcluster)


#there are two methods can be used ---- 'KMeans', 'SpectralClustering', 'AgglomerativeClustering'

#choose method
method = 'DBSCAN'


if method == 'KMeans':

    k_instance_kmean1 = \
    KMeans(max_iter=200, n_clusters = n_clusters1, random_state=0, init='k-means++').fit(pixel_position1)
    k_instance_kmean2 = \
    KMeans(max_iter=100, n_clusters = n_clusters2, init='k-means++').fit(pixel_position2)

    cluster_label1 = k_instance_kmean1.labels_
    cluster_label2 = k_instance_kmean2.labels_
    cluster_num1 = k_instance_kmean1.cluster_centers_.shape[0]
    cluster_num2 = k_instance_kmean2.cluster_centers_.shape[0]


if method == "AgglomerativeClustering":

    cluster_agg1 = AgglomerativeClustering(n_clusters = n_clusters1).fit(pixel_position1)
    cluster_agg2 = AgglomerativeClustering(n_clusters = n_clusters2).fit(pixel_position2)

    cluster_label1 = cluster_agg1.labels_
    cluster_label2 = cluster_agg2.labels_
    cluster_num1 = cluster_label1.max()
    cluster_num2 = cluster_label2.max()


if method == "DBSCAN":

    cluster_DBSCAN1 = DBSCAN(eps=eps1, min_samples=mini1).fit(pixel_position1)
    cluster_DBSCAN2 = DBSCAN(eps=eps2, min_samples=mini2).fit(pixel_position2)

    cluster_label1 = cluster_DBSCAN1.labels_
    cluster_label2 = cluster_DBSCAN2.labels_

    cluster_num1 = cluster_label1.max()
    cluster_num2 = cluster_label2.max()
if method == 'SpectralClustering':

    spectral_model_rbf1 = SpectralClustering(n_clusters = n_clusters1, affinity ='rbf')
    spectral_model_rbf2 = SpectralClustering(n_clusters = n_clusters2, affinity ='rbf')

    labels_rbf1 = \
    spectral_model_rbf1.fit_predict(pixel_position1)
    labels_rbf2 = \
    spectral_model_rbf2.fit_predict(pixel_position2)

    cluster_label1 = labels_rbf1
    cluster_label2 = labels_rbf2
    cluster_num1 = labels_rbf1.max()
    cluster_num2 = labels_rbf2.max()


#cluster
cluster_dict1, cluster_num_new1 = pixel_cluster2(cluster_label1, pixel_position1, cluster_num1, 15)
cluster_dict2 = pixel_cluster(cluster_label2, pixel_position2, cluster_num2)

cluster_num1 = cluster_num_new1


#find the vertices
right_down_dict1, right_up_dict1, left_down_dict1, left_up_dict1 \
= cluster_vertex(cluster_num1, cluster_dict1)
right_down_dict2, right_up_dict2, left_down_dict2, left_up_dict2 \
= cluster_vertex(cluster_num2, cluster_dict2)


#find clusters angles
cluster_angles1 = cluster_angle_fun(cluster_num1, cluster_dict1, \
                                    right_down_dict1, right_up_dict1, \
                                    left_down_dict1, left_up_dict1)


cluster_angles2 = cluster_angle_fun(cluster_num2, cluster_dict2, \
                                    right_down_dict2, right_up_dict2, \
                                    left_down_dict2, left_up_dict2)


#find fibers angles
tol_in_cluster1 = tol_deg_in_cluster1*pi/180
tol_out_cluster1 = tol_deg_in_cluster1*pi/180
tol_in_cluster2 = tol_deg_in_cluster2*pi/180
tol_out_cluster2 = tol_deg_in_cluster2*pi/180
```

Cluster which has a higher height than this, will be rolled out.

```
#print(cluster_num1)

cluster_dict_new1, right_down_dict_new1, right_up_dict_new1, \
left_down_dict_new1, left_up_dict_new1 \
= cluster_update(cluster_num1, cluster_angles1, tol_in_cluster1, tol_out_cluster1, \
                 cluster_dict1, right_down_dict1, right_up_dict1, \
                 left_down_dict1, left_up_dict1)

cluster_dict_new2, right_down_dict_new2, right_up_dict_new2, \
left_down_dict_new2, left_up_dict_new2 \
= cluster_update(cluster_num2, cluster_angles2, tol_in_cluster2, tol_out_cluster2, \
                 cluster_dict2, right_down_dict2, right_up_dict2, \
                 left_down_dict2, left_up_dict2)



cluster_angle_new1 = cluster_angle_fun(cluster_num1, cluster_dict_new1, \
                                       right_down_dict_new1, right_up_dict_new1, \
                                       left_down_dict_new1, left_up_dict_new1)

cluster_angle_new2 = cluster_angle_fun(cluster_num2, cluster_dict_new2, \
                                       right_down_dict_new2, right_up_dict_new2, \
                                       left_down_dict_new2, left_up_dict_new2)



cluster_dict_new_posi1, cluster_dict_new_nega1, right_down_dict_posi1, right_up_dict_posi1, \
left_down_dict_posi1, left_up_dict_posi1, right_down_dict_nega1, right_up_dict_nega1, \
left_down_dict_nega1, left_up_dict_nega1\
= cluster_group(cluster_num1, cluster_dict_new1, cluster_angle_new1, \
    right_down_dict_new1, right_up_dict_new1, \
                left_down_dict_new1, left_up_dict_new1)

cluster_dict_new_posi2, cluster_dict_new_nega2, right_down_dict_posi2, right_up_dict_posi2, \
left_down_dict_posi2, left_up_dict_posi2, right_down_dict_nega2, right_up_dict_nega2, \
left_down_dict_nega2, left_up_dict_nega2 \
= cluster_group(cluster_num2, cluster_dict_new2, cluster_angle_new2, \
    right_down_dict_new2, right_up_dict_new2, \
                left_down_dict_new2, left_up_dict_new2)
```

```
## Selelct the muscle fibers

#define the angle sets that will be used for upper pic

cluster_dict_new1, right_down_dict1, right_up_dict1, left_down_dict1, left_up_dict1 = \
cluster_dict_new_nega1, right_down_dict_nega1, right_up_dict_nega1, \
left_down_dict_nega1, left_up_dict_nega1

#define the angle sets that will be used for downner pic
cluster_dict_new2, right_down_dict2, right_up_dict2, left_down_dict2, left_up_dict2 = \
cluster_dict_new_posi2, right_down_dict_posi2, right_up_dict_posi2, \
left_down_dict_posi2, left_up_dict_posi2


value_dict1 = Value_Funtion(1, w1_1, w2_1, image_new_clean1, \
                            cluster_dict_new1, right_down_dict1, right_up_dict1, \
                            left_down_dict1, left_up_dict1)

value_dict2 = Value_Funtion(2, w1_2, w2_2, image_new_clean2, \
                            cluster_dict_new2, right_down_dict2, right_up_dict2, \
                            left_down_dict2, left_up_dict2)
```

Still take the upper image (contains the fascicle) as an example:

Show the Clusters

In [17]:
```
for i in range(cluster_num1):
    if i not in cluster_dict1:
        continue
    image_cluster1 = show_cluster(image1, cluster_dict1[i])
    #plt.savefig("image_cluster1.jpg")
    plt.show()
    plt.figure(figsize = (6, 25))
    plt.subplot(cluster_num1, 1, i+1)
    plt.imshow(image_cluster1)
```

There are some clusters we obtained using DBSCAN.

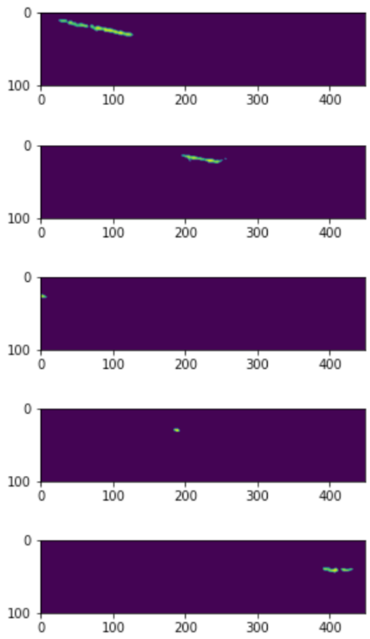

If we recluster them, we will have:

```
In [133]: for i in cluster_dict_new1.keys():
              image_cluster_new1 = show_cluster(image1, cluster_dict_new1[i]
              plt.figure(figsize = (12, 25))
              plt.subplot(cluster_num1, 1, i+1)
              plt.imshow(image_cluster_new1)
```

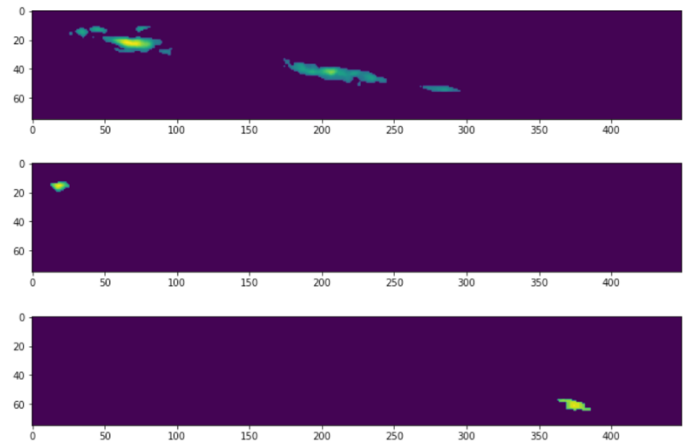

In this case, we can assume that these parameters are good because a line-like structure shows in one cluster, which might be the fascicle. Then, we just need to select weights for the value function to assign a value to each cluster. The cluster which has the highest value will be selected as the fascicle.

10. Filters

In 9, we used a set of parameters to get some clusters and one of them may represent the fascicle. However, in the real applications, we do not have the chance to work the

parameters out step by step. Therefore, we may need to guess. If we only have one shot and the guess is not good, the result may not be satisfactory. Therefore, we employ multiple sets of parameters to perform the detection. Then we can take the weighted average on the results obtained using all the sets of parameters. The weights are computed depending on the previous results, i.e., if the new result (from one set of parameters) is too far away from the previous one, the weight is set to be low, vice versa. In fact, we can also use the historical data (i.e., the viscous function mentioned in the paper) obtained from completed experiments.

For example:
Here case_number is the number of the sets of parameters.

```
for n in range(case_number+1):

    if n == 0:
        b1 = 90
        b2 = 90
        eps1 = 4.5
        eps2 = 5.5
        mini1 = 20
        mini2 = 50

        tol_deg_in_cluster1 = 3
        tol_deg_out_cluster1 = 3
        tol_deg_in_cluster2 = 5
        tol_deg_out_cluster2 = 5

        w1_1 = 0.2
        w2_1 = 50

        w1_2 = 0.2
        w2_2 = 200
        limit1 = 25
        limit2 = 100
        #augment the image

        #0
        right_pixel1_last, left_pixel1_last, right_pixel2_last, left_pixel2_last \
        = np.array([71, 461]), np.array([11, 1]), np.array([41, -11]), np.array([-11, 509])

        image_augment1 = fs.ellipse_augmentation7(image1, right_pixel1_last, left_pixel1_last, \
                                                  0.00250, 0.0075, 0.00925, 3.75, 3.25, 2.00)

        image_augment2 = fs.ellipse_augmentation7(image2, right_pixel2_last, left_pixel2_last, \
                                                  0.000550, 0.001, 0.0075, 2.75, 2.5, 1.75)



    elif n == 1:
        b1 = 80
        b2 = 80
        eps1 = 4.5
        eps2 = 5.5
        mini1 = 40
        mini2 = 10
        tol_deg_in_cluster1 = 2.75
        tol_deg_out_cluster1 = 2.75
        tol_deg_in_cluster2 = 5
        tol_deg_out_cluster2 = 5
        w1_1 = 0.2
        w1_2 = 0.2
        w2_1 = 10
        limit1 = 30
        limit2 = 40
        w2_2 = 200


        #1
        right_pixel1_last, left_pixel1_last, right_pixel2_last, left_pixel2_last \
        = np.array([76, 311]), np.array([31, 1]), np.array([31, -11]), np.array([-11, 509])

        image_augment1 = fs.ellipse_augmentation7(image1, right_pixel1_last, left_pixel1_last, \
                                                  0.00250, 0.0075, 0.00925, 3.75, 3.25, 2.00)

        image_augment2 = fs.ellipse_augmentation7(image2, right_pixel2_last, left_pixel2_last, \
                                                  0.000550, 0.001, 0.0075, 2.75, 2.5, 1.75)
```

```
elif n == 2:
    b1 = 85
    b2 = 95
    eps1 = 4.5
    eps2 = 5.5
    mini1 = 20
    mini2 = 40

    tol_deg_in_cluster1 = 3
    tol_deg_out_cluster1 = 3
    tol_deg_in_cluster2 = 5
    tol_deg_out_cluster2 = 5

    w1_1 = 0.2
    w2_1 = 50

    w1_2 = 0.2
    w2_2 = 200
    limit1 = 25
    limit2 = 100
    #2
    right_pixel1_last, left_pixel1_last, right_pixel2_last, left_pixel2_last \
    = np.array([66, 311]), np.array([1, 11]), np.array([31, -11]), np.array([-11, 509])

    image_augment1 = fs.ellipse_augmentation7(image1, right_pixel1_last, left_pixel1_last, \
                                       0.00250, 0.0075, 0.00925, 3.75, 3.25, 2.00)

    image_augment2 = fs.ellipse_augmentation7(image2, right_pixel2_last, left_pixel2_last, \
                                       0.000550, 0.001, 0.0075, 2.75, 2.5, 1.75)


elif n == 3:
    b1 = 95
    b2 = 95
    limit1 = 30
    limit2 = 45
    eps1 = 4.5
    eps2 = 5.5
    mini1 = 20
    mini2 = 50
    tol_deg_in_cluster1 = 3
    tol_deg_out_cluster1 = 3
    tol_deg_in_cluster2 = 5
    tol_deg_out_cluster2 = 5
    w1_1 = 0.2
    w2_1 = 50
    w1_2 = 0.2
    w2_2 = 200

    #3
    right_pixel1_last, left_pixel1_last, right_pixel2_last, left_pixel2_last \
    = np.array([86, 171]), np.array([46, -11]), np.array([36, -11]), np.array([-11, 509])

    image_augment1 = fs.ellipse_augmentation7(image1, right_pixel1_last, left_pixel1_last, \
                                       0.0150, 0.0175, 0.02525, 3.75, 3.25, 2.00)

    image_augment2 = fs.ellipse_augmentation7(image2, right_pixel2_last, left_pixel2_last, \
                                       0.000550, 0.001, 0.0075, 2.75, 2.5, 1.75)
```

also have several cases……

```
    else:
        b1 = 90
        b2 = 80
        image_augment1 = image1
        image_augment2 = image2


    #there are two methods can be used ---- 'KMeans', 'SpectralClustering', 'AgglomerativeClustering', 'DBSCAN'




    try:
        angle_max1, angle_max2 = \
        fs.fiber_detector_s4('DBSCAN', b1, b2, connectivity1, connectivity2, mini1, mini2, \
                           eps1, eps2, tol_deg_in_cluster1, tol_deg_out_cluster1, \
                           tol_deg_in_cluster2, tol_deg_out_cluster2, \
                           w1_1, w2_1, w1_2, w2_2, image_augment1, image_augment2, limit1, limit2)


        r1 = fs.viscosity_weight(angle_max1, last_angle1)
        r2 = fs.viscosity_weight(angle_max2, last_angle2)
        print('filter' + str(n) + ' worked')
        #print(angle_max1, r1, angle_max2, r2)
    except:
        print('filter' + str(n) + ' has compilling error')
        angle_max1 = last_angle1
        angle_max2 = last_angle2
        r1 = 0.00000001
        r2 = 0.00000001


    angle_weighted1 = angle_max1 * r1
    angle_weighted2 = angle_max2 * r2


    angle_weighted_sum1 = angle_weighted_sum1 + angle_weighted1
    w1 = w1 + r1
    angle_weighted_sum2 = angle_weighted_sum2 + angle_weighted2
    w2 = w2 + r2

angle_weighted_ave1 = angle_weighted_sum1/(w1+0.0000000001)
angle_weighted_ave2 = angle_weighted_sum2/(w2+0.0000000001)

final_angle1 = fs.viscosity_process(angle_weighted_ave1, last_angle1)
final_angle2 = fs.viscosity_process(angle_weighted_ave2, last_angle2)


final_angle = final_angle1 + final_angle2
print('final_angle: %2f'   %(final_angle))
aug_visc.append(final_angle)
```

They are called viscosity function, but they are used to compute the weights here.

How to Find the Ground Truth

The human annotation, which represents the ground truth (see the picture below), can be extracted using the following methods.

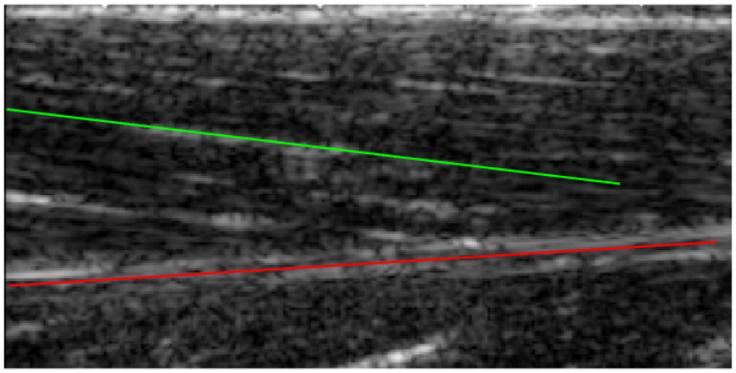

Fig: Human annotated fascicle (green) and aponeurosis (red).

To extract the green and red lines from the labeled US images, we designed the following code.

```
#extract the red label
def extract_red(image):
    image_r = np.zeros((image.shape[0],image.shape[1],3), np.uint8)

    for i in range(image.shape[0]):
        for j in range(image.shape[1]):
            b,g,r = image[i,j]
            if((r-b)>40 and (r-g)>40):
                b=0
                g=0
                r=0

            else:
                b=255
                g=255
                r=255

            image_r[i,j]=[r,g,b]

    return image_r
#extract the green label
def extract_green(image):
    image_g = np.zeros((image.shape[0],image.shape[1],3), np.uint8)

    for i in range(image.shape[0]):
        for j in range(image.shape[1]):
            b,g,r = image[i,j]
            if(g-b>40 and g-r>40):
                b=0
                g=0
                r=0

            else:
                b=255
                g=255
                r=255

            image_g[i,j]=[r,g,b]

    return image_g
```

The result should look like this:

```
In [137]: imagenew = fs.extract_green(image1)
          plt.imshow(imagenew)

Out[137]: <matplotlib.image.AxesImage at 0x7fa9c3597190>
```

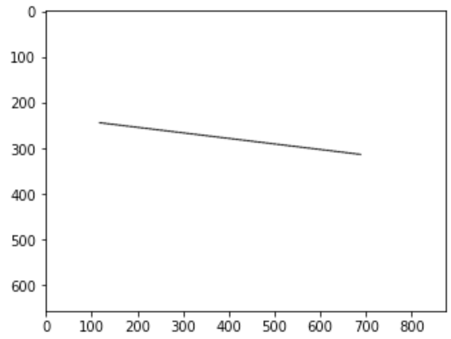


```
In [138]: imagenew = fs.extract_red(image1)
          plt.imshow(imagenew)

Out[138]: <matplotlib.image.AxesImage at 0x7fa9c3eb4a90>
```

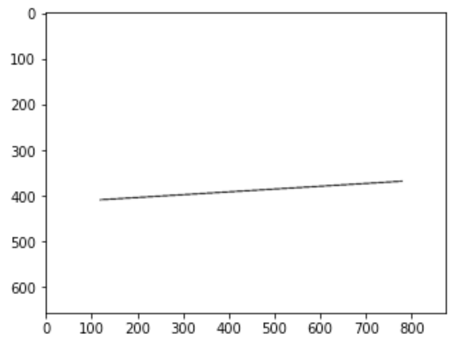

To compute the orientation of the extracted line, we just need to run the following code.

```
In [51]: y_left = 300
         y_right = 400
         x_left = 0
         x_right = 0

         for i in range(imagenew.shape[0]):
             if imagenew[i][y_left][0] < 10:
                 x_left = i
                 break

         for i in range(imagenew.shape[0]):
             if imagenew[i][y_right][0] < 10:
                 x_right = i
                 break

In [59]: np.arctan((x_right - x_left)/(y_right - y_left))*180/pi

Out[59]: 6.84277341263094
```

In this example, the ground truth orientation is around 6.8.

We can also define them as functions:

```
def upper_extract(image):
    y_left = 140
    y_right = 650
    x_left = 0
    x_right = 0

    image_up = fs.extract_green(image)

    for i in range(image_up.shape[0]):
        if image_up[i][y_left][0] < 10:
            x_left = i
            break
    #print('x_left: %2f' %(x_left))

    for i in range(image_up.shape[0]):
        if image_up[i][y_right][0] < 10:
            x_right = i
            break
    #print('x_right: %2f' %(x_right))

    return np.arctan((x_right - x_left)/(y_right - y_left))*180/pi


def lower_extract(image):
    y_left = 140
    y_right = 710
    x_left = 0
    x_right = 0

    image_up = fs.extract_red(image)

    for i in range(image_up.shape[0]):
        if image_up[i][y_left][0] < 10:
            x_left = i
            break
    #print('x_left: %2f' %(x_left))

    for i in range(image_up.shape[0]):
        if image_up[i][y_right][0] < 10:
            x_right = i
            break
    #print('x_right: %2f' %(x_right))

    return np.arctan((x_right - x_left)/(y_right - y_left))*180/pi
```

Then, we can get all ground truth orientation from one trial. Let's take A2 Angle 15 Trial 1 as an example:

```
ref_up_vec = []
ref_down_vec = []
ref_vec = []

for i in range(15):
    image1 = cv2.imread('./data/Ankle_A02/angle_15_trial1/Labeled/TA' + str(i+1) + '.tif')
    ref_up = upper_extract(image1)
    ref_down = lower_extract(image1)

    ref_up_vec.append(ref_up)
    ref_down_vec.append(ref_down)
    ref_vec.append(ref_up-ref_down)
```

Then, the ground truth can be obtained easily.
